# Evidence for Prehistoric Origins of Egyptian Mummification in Late Neolithic Burials

**DOI:** 10.1371/journal.pone.0103608

**Published:** 2014-08-13

**Authors:** Jana Jones, Thomas F. G. Higham, Ron Oldfield, Terry P. O'Connor, Stephen A. Buckley

**Affiliations:** 1 Department of Ancient History, Faculty of Arts, Macquarie University, Sydney, New South Wales, Australia; 2 Oxford Radiocarbon Accelerator Unit, Research Laboratory for Archaeology and the History of Art, University of Oxford, Oxford, United Kingdom; 3 Department of Biological Sciences, Faculty of Science, Macquarie University, Sydney, New South Wales, Australia; 4 Department of Archaeology, University of York, The Kings Manor, York, United Kingdom; 5 BioArch, Departments of Archaeology, Biology and Chemistry (S-Block), University of York, York, United Kingdom; Museo Nazionale Preistorico Etnografico ‘L. Pigorini’, Italy

## Abstract

Traditional theories on ancient Egyptian mummification postulate that in the prehistoric period (i.e. the Neolithic and Chalcolithic periods, 5^th^ and 4^th^ millennia B.C.) bodies were naturally desiccated through the action of the hot, dry desert sand. Although molding of the body with resin-impregnated linen is believed to be an early Pharaonic forerunner to more complex processes, scientific evidence for the early use of resins in artificial mummification has until now been limited to isolated occurrences during the late Old Kingdom (c. 2200 B.C.), their use becoming more apparent during the Middle Kingdom (c. 2000-1600 BC). We examined linen wrappings from bodies in securely provenanced tombs (pit graves) in the earliest recorded ancient Egyptian cemeteries at Mostagedda in the Badari region (Upper Egypt). Our investigations of these prehistoric funerary wrappings using a combination of gas chromatography-mass spectrometry (GC-MS) and thermal desorption/pyrolysis (TD/Py)-GC-MS have identified a pine resin, an aromatic plant extract, a plant gum/sugar, a natural petroleum source, and a plant oil/animal fat in directly AMS-dated funerary wrappings. Predating the earliest scientific evidence by more than a millennium, these embalming agents constitute complex, processed recipes of the same natural products, in similar proportions, as those utilized at the zenith of Pharaonic mummification some 3,000 years later. The antibacterial properties of some of these ingredients and the localized soft-tissue preservation that they would have afforded lead us to conclude that these represent the very beginnings of experimentation that would evolve into the famous mummification practice of the Pharaonic period.

## Introduction

No chemical investigations or analyses of the organic compounds present in funerary wrappings of the prehistoric period (c. 4500 – 3350 BC) have ever been reported in the literature, nor has their presence as early as the fifth millennium BC been previously proposed by others [1]. It has been assumed that preservation of soft tissues was predominantly through natural processes afforded by the favorable burial environment, rather than by the complex and deliberate physico-chemical intervention that characterizes the mummification of later times [2], [3]. The Old Kingdom is generally regarded as the start of true Egyptian mummification c. 2500 BC [4], with the utilization of preservative resins becoming more evident by the Middle Kingdom (c. 2000 – 1600 BC) [5]. Where resins with preservative qualities have been identified in the embalming agents, the mummies have generally been of a relatively late date [6], [7], [8], [9], [10], [11], although a conifer resin has been identified in one mummy from the late Old Kingdom (c. 2200 BC) [12].

Modern investigative chemical techniques applied to securely provenanced and dated mummies and embalming material [6], [8], [12], [13], [14], [15] have provided insights into the organic materials used in mummification during Egypt's Pharaonic period (c. 2900 – 332 BC) [16]. Yet there have been no such studies prior to this period.

Here we present the first chemical investigation of directly AMS-dated linen funerary wrappings, skin and ‘reed’ matting material from bodies in securely provenanced Badarian (Late Neolithic) and Predynastic (Chalcolithic) period tombs (pit graves) at Mostagedda in the Badari region, Upper Egypt (c. 4500 BC – 3350 BC [16]). Specifically, analysis was undertaken on textile wrappings impregnated with ‘resin’ (*sensu lato*), which is regarded as the main component of early Pharaonic attempts at corporeal preservation before the later introduction (c. 2500 BC) of a desiccant (natron) and evisceration [17].

Samples of textiles from cemeteries at Badari and Mostagedda were sent to the Chadwick (now Bolton) Museum in Bolton, UK by the excavators in the early 20^th^ century [18], [19]. The site of Badari gave its name to the earlier cultural phase (c. 4500 – 3700 BC [16], [20], also referred to as the Late Neolithic period), which in this region preceded the Naqada culture (i.e., the Predynastic/Chalcolithic period) beginning c. 3800/3700 BC [16], [20]. Both phases occur in the cemeteries at the two sites.

Microscopical analysis by Jones at the later cemetery HK43 at Hierakonpolis (Predynastic period, Naqada IIA-C, c. 3600 – 3400 BC), had confirmed the presence of a ‘resinous’ substance permeating thick layers of linen firmly wrapped around parts of a number of bodies, most notably the back of the head, the jaw and the hands [21]. These observations were based on physical appearance only, not on biochemical analyses. Consequently, the nature and composition, and therefore significance of these amorphous organic residues at Hierakonpolis necessarily remains unknown. Yet the pattern of textile use was reminiscent of early reports of Badarian period inhumations at the site of Badari, which mention seven cases in which the head was wrapped in textile and one example of a pad of textile at the hands [18].

The possibility of the same, or a similar, anthropogenic process used in the earliest documented Egyptian burials prompted scientific investigation of this older material held in Bolton Museum [1]. Nine samples of wrappings from Badari and 42 from Mostagedda were examined microscopically for traces of ‘resins’ (Figures S1–S12 in Appendix S1). Badarian and Predynastic samples from Mostagedda were selected for chemical investigation because of the greater quantity available, and in order to give a time series from a single site.

Characteristic artifacts (pottery, stone palettes), and the location of the tombs in the cemeteries, provide sound archaeological evidence for the relative dating of the burials [19], [22] (Table S1 in Appendix S1). The spin direction of the yarn from which the textiles were woven is also a contributing factor. A major technological change in the direction of spin from the ‘Z’ direction to the ‘S’ occurred in the early Predynastic period; extant Egyptian textiles from early Naqada IB (c. 3700/3600 BC) onward are woven from ‘S’ twist yarn [23].

**Figure 1:**
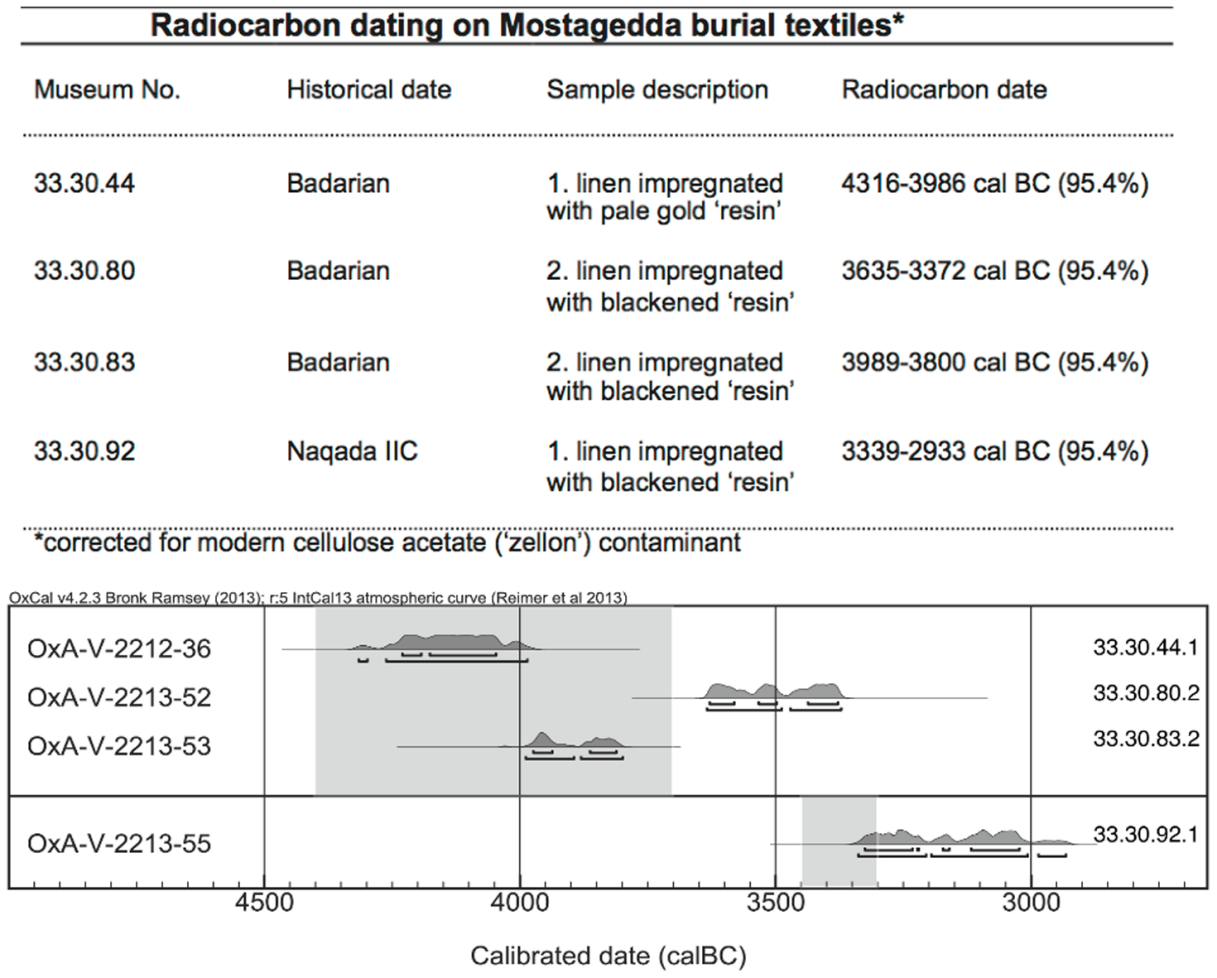
Calibrated ages for the corrected textile dated samples. The table displays the data corresponding to these probability distributions at 68.2% and 95.4% probability. The shaded areas denote the accepted archaeological age range for the Badarian (top of picture) and Naqada IIC period (bottom) respectively. (Figure generated using OxCal 4.1 (Bronk Ramsey 2009)).

Radiocarbon dating was undertaken on a number of these samples (Fig. 1) to provide calendrical calibration of the significant archaeological evidence for these Mostagedda burials. A combination of gas chromatography-mass spectrometry (GC-MS) and thermal desorption/pyrolysis (TD/Py)-GC-MS facilitates the molecular separation, characterization and identification of both the free (solvent extractable) biomarker compounds, and the recognizable sub-units of polymeric materials not amenable to the more conventional GC-MS approach [14].

## Methods

### Microscopy

**Figure 2:**
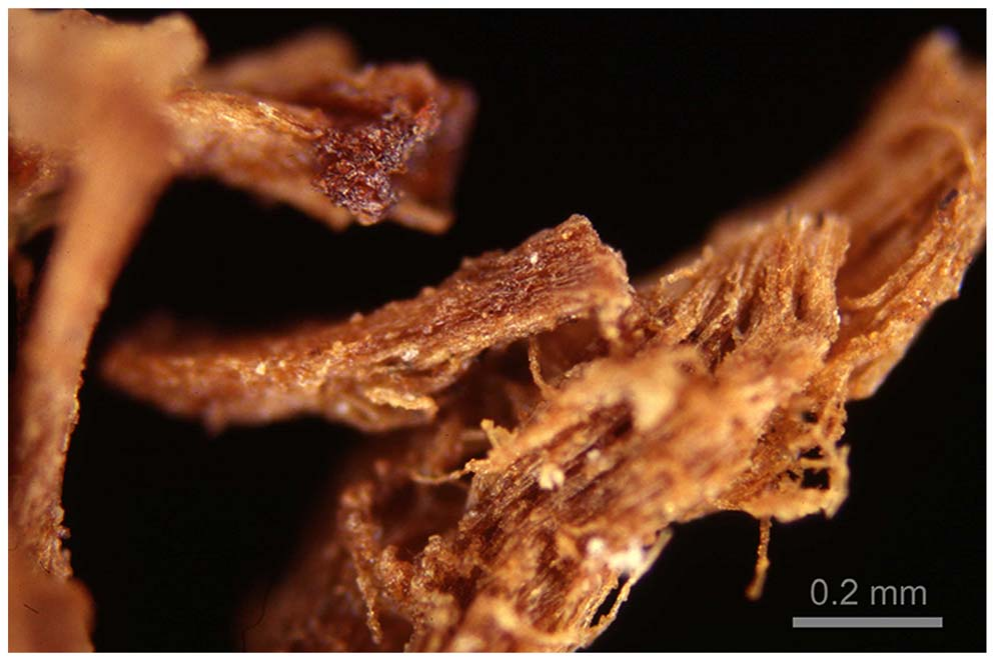
33.30.44 Tomb No. 3538, Badarian Period, Mostagedda. The most convincing depiction of the ‘toffee-like’ (presumed) ‘resin’ was effected in the light microscope using HD illumination.

Textile specimens in the Bolton Museum were initially examined at low magnification with a Leica MZ6 stereomicroscope, and those that presented themselves likely to have ‘resin’ were photographed macroscopically and then through the stereomicroscope, with incident twin-armed fiberoptic illumination [24]. Further examination was carried out in the microscopy unit in the Department of Biological Sciences, Macquarie University, Sydney, Australia. The most convincing depiction of the ‘toffee-like’ (presumed) resin was effected in the light microscope using Epiplan HD (Hellfeld/Dunkelfeld = Brightfield/Darkfield) objectives on a Zeiss Universal microscope [25] (Fig. 2).

Darkfield epi-illumination techniques require very strong illumination but give an image with brilliant contrast well-suited to very high quality colour photography. There was no heat damage to the specimen, but the major disadvantage is the very limited and virtually uncontrollable depth of field. A rotatable sector stop in the light path will reduce the intensity but promote the possibility of adding controlled relief to the image.

Investigations involved the excision of a piece of yarn 3 to 4 mm in length, which was placed in a drop of glycerine on a 3″×1″ microscope slide. The ultimate (individual) flax fibres were carefully teased apart with a pair of mounted needles, and covered with a No. 1 cover slip, carefully avoiding air bubbles. Observation with transmitted light illumination (Brightfield/Crossed polars) showed that the resinous substances had been incorporated into the cell walls.

### Dating

Samples of textile were dated at the Oxford Radiocarbon Accelerator Unit (ORAU), University of Oxford. The samples were treated with a dilute acid wash (0.5 M HCl at RT for 30 mins), rinsed and dried. Combustion of the treated samples was achieved using a Europa Scientific ANCA-MS system, comprising a 20-20 IR mass spectrometer linked with a Roboprep or Carlo-Erba CHN sample converter unit. This operates in a continuous flow mode with a He carrier gas. δ^13^C values are reported with reference to VPDB. Graphitisation of CO_2_ was achieved using the standard ORAU method that is outlined in Dee and Bronk Ramsey [26]. The Oxford AMS radiocarbon instrumentation is described by Bronk Ramsey [27]. Radiocarbon dates are expressed as conventional radiocarbon ages BP after Stuiver and Polach [28]. The determinations obtained were corrected based on the quantified proportions of modern and ^14^C-free exogenous carbon in them derived from conservation attempts on the textiles (see Appendix S1).

### Chemical Analyses

The amorphous organic residues were chemically characterized and identified using a dual analytical approach of conventional gas chromatography (GC-MS) and sequential thermal desorption-gas chromatography-mass spectrometry (TD-GC-MS) and pyrolysis-gas chromatography-mass spectrometry (Py-GC-MS), thereby allowing the chemical analysis of both free and bound/polymerized biomarkers likely to be present [14].

The samples for GC and GC-MS were initially ground to a fine powder. A weighed amount of these ground samples (from ∼0.5–25 mg depending on sample available) was taken, and where appropriate an internal standard was added for quantification (10–100 µg of tetratriacontane, *n*-C_34_ alkane). These samples were then extracted with an appropriate volume (0.1–1 mL) of chloroform-methanol solution (2∶1 v/v; 3×60 min sonication). After centrifugation (20 min, 1000 rpm) the supernatant solvent was removed from the residue and placed in a vial. The three extracts were combined and the solvent reduced by rotary evaporation. Following transfer of the combined extracts to a screw-capped vial, the remaining solvent was removed by evaporation under a gentle stream of nitrogen at 40°C. The residue was reweighed to give total lipid extracts (TLE). The TLEs were trimethylsilylated using *N,O*-bis(trimethylsilyl)trifluoroacetamide (Sigma-Aldrich Chemical Co., St Louis, MO, USA) containing 1% of trimethylchlorosilane (30–50 µl, 70°C, 1 hour). Excess BSTFA was then removed under a gentle stream of nitrogen and the derivatized sample redissolved in dichloromethane and analyzed by GC and GC-MS. Identification of compounds was achieved on the basis of both their mass spectra (NIST Mass Spectral Database and additional data referenced in the Appendix S1), and retention times (see Appendix S1), and the analysis of reference samples.

The samples for sequential TD-GC-MS and Py-GC-MS were initially ground to a fine powder prior to analysis. A weighed amount of these ground samples (0.1–1 mg of sample weighed using a microanalytical balance) was taken and these samples loaded into quartz tubes before being inserted into the pyrolysis probe for TD/Py-GC-MS. The samples were thermally desorbed at 310°C, followed by pyrolysis at 610°C. The TD/Py temperature was held for 10 s. Identification of compounds was achieved on the basis of both their mass spectra (NIST Mass Spectral Database and additional data referenced in Appendix S1), and retention times (see Appendix S1), and the analysis of reference samples.

## Results and Discussion

The results of this study are summarized in Table 1. The amount of organic residue – ‘resin’ (*sensu lato*) – extracted from the samples varied according to the type of material analyzed (Table 1). As might be expected, two of the reed matting samples yielded little extractable organic material (<1%), with any ‘resin’ likely to derive from limited contact with the impregnated textiles from the bodies (although see results on the aromatic plant extract below). One reed matting sample did contain an appreciable amount of extractable ‘resin’ (28%), likely to be explained by the particularly ‘shiny’ nature of this sample, suggesting it had a coating of the ancient ‘resin’ recipe on its surface. In contrast, the ‘resin’ sample analyzed was largely solvent soluble (56%), reflecting its nature as an amorphous organic residue. The organic extract from the skin sample constituted 18% of the total sample, which is consistent with ‘resin’ adhering to the surface of the body, as might be expected, and possibly a component from undegraded human skin lipids. Of the ‘resin’-impregnated textile samples analyzed, where the majority of the sample would be expected to be the cellulose-based linen, the ancient ‘resin’ component constituted 2–13%, with an average of 7%. The abundance of ancient ‘resin’ in these samples correlates with what would be expected in each of these types of materials, providing a sound archaeological context for a meaningful scientific investigation, with a particular focus on the ‘resin’ impregnating the textiles used to wrap around the bodies in these burials.

**Table 1:** Mostagedda ‘mummy’ textiles, origin of balm samples, the abundance of ancient ‘resin’ in the textiles, and their chemical composition.

Museum and specimen No.^1^	Tomb No.	Historical date	Sample location* and description	Ancient ‘resin’^2^ (%)^3^	Plant oil/animal fat (%)	Plant Wax (%)	Pine resin (%)	Aromatic plant extract (%)	Sugar/gum (%)	Natural petroleum seep (%)	Inferred components of funerary balms	Relative abundance (%)^3^
Female adult 33.30.44 2	3538	Badarian	‘cloth near knees’* impregnated with pale gold ‘resin’	9	80	-	9	10	1	0.2	Plant oil/animal fat	80
											Pine resin	9
											Aromatic plant extract	10
											Sugar/gum	1
											Natural petroleum	0.2
Female adult 33.30.44 3	3538	Badarian	reed matting	<1	34	7	2	54	1	2	Plant oil/animal fat	34
											Plant wax	7
											Pine resin	2
											Aromatic plant extract	54
											Sugar/gum	1
											Natural petroleum	2
Female adult 33.30.72 1	494	Badarian	linen ‘on body’* impregnated with blackened ‘resin’	2	72	-	0.9	19	1	7	Plant oil/animal fat	72
											Pine resin	0.9
											Aromatic plant extract	19
											Sugar/gum	1
											Natural petroleum	7
Male adult 33.30.80 1	1215	Badarian	‘resinous’ matting	<1	70	5	0.3	24	0.2	0.4	Plant oil/animal fat	70
											Plant wax	5
											Pine resin	0.3
											Aromatic plant extract	24
											Sugar/gum	0.2
											Natural petroleum	0.4
Male adult 33.30.80 2	1215	Badarian	linen impregnated with ‘resin’ (brown)	13	80	0.2	2.3	17	0.6	trace	Plant oil/animal fat	80
											Plant wax	0.2
											Pine resin	2.3
											Aromatic plant extract	17
											Sugar/gum	0.6
											Natural petroleum	trace
Male adult 33.30.80 3	1215	Badarian	‘resin’	56	86	0.5	0.1	13	0.4	trace	Plant oil/animal fat	86
											Plant wax	0.5
											Pine resin(?)	0.1
											Aromatic plant extract	13
											Sugar/gum	0.4
											Natural petroleum	trace
Male adult 33.30.80 4	1215	Badarian	linen impregnated with blackened ‘resin’	8	67	2	11	15	4	1	Plant oil/animal fat	67
											Plant wax	2
											Pine resin	11
											Aromatic plant extract	15
											Sugar/gum	4
											Natural petroleum	1
Male adult 33.30.80 6	1215	Badarian	linen impregnated with blackened ‘resin’	11	81	0.3	2	14	2	0.3	Plant oil/animal fat	81
											Plant wax	0.3
											Pine resin	2
											Aromatic plant extract	14
											Sugar/gum	2
											Natural petroleum	0.3
Female adult 33.30.83 2	1214	Badarian	linen impregnated with blackened ‘resin’	10	79	-	0.6	20	0.3	-	Plant oil/animal fat	79
											Pine resin	0.6
											Aromatic plant extract	20
											Sugar/gum	0.3
Female adult 33.30.83 3	1214	Badarian	reed matting (shiny)	28	62	0.2	0.4	36	0.6	-	Plant oil/animal fat	62
											Plant wax	0.2
											Pine resin	0.4
											Aromatic plant extract	36
											Sugar/gum	0.6
Female adult 33.30.83 4	1214	Badarian	skin	18	88	-	0.5	11	0.1	-	Plant oil/animal fat	88
											Pine resin	0.5
											Aromatic plant extract	11
											Sugar/gum	0.1
Female(?) adult 33.30.30 1	11725	Naqada IIB	linen impregnated with blackened ‘resin’	2	66	-	3.5	11	trace	19	Plant oil/animal fat	66
											Pine resin	3.5
											Aromatic plant extract	11
											Sugar/gum	trace
											Natural petroleum	19
Male adult 33.30.53 1	1609	Naqada IIC	‘entire body covered’* in linen impregnated with blackened ‘resin’	1	76	-	1.5	5	1	16	Plant oil/animal fat	76
											Pine(?) resin	1.5
											Aromatic plant extract	5
											Sugar/gum	1
											Natural petroleum	16
Male adult 33.30.59 1	1640	Predynastic	linen impregnated with blackened ‘resin’	8	95	-	1	2.4	1	0.6	Plant oil/animal fat	95
											Pine resin	1
											Aromatic plant extract	2.4
											Sugar/gum	1
											Natural petroleum	0.6
Female adult 33.30.92 1	1637	Naqada IIC	‘in front of body’* linen impregnated with blackened ‘resin’	9	79	-	2.2	17	1.5	0.3	Animal fat	79
											Pine resin	2.2
											Aromatic plant extract	17
											Sugar/gum	1.5
											Natural petroleum	0.3
Female adult 33.30.92 2	1637	Naqada IIC	‘in front of body’* linen impregnated with blackened ‘resin’	3	64	5	10	18	1	1.5	Plant oil/animal fat	64
											Plant wax	5
											Pine resin	10
											Aromatic plant extract	18
											Sugar/gum	1
											Natural petroleum	1.5

1 All samples in this study were from Bolton Museum.

2 The term ‘resin’ denotes physical appearance and does not presuppose any chemical composition or biological origin.

3 Percentage of the extracted ancient ‘resin’, i.e. ‘balms’, was quantified by the weight of extract minus the Zellon component (determined by GC and GC/MS; also see radiocarbon SI), and, where sample sizes were sufficient (see text), internal standards added at the extraction stage. Percentage relative abundance of components in extracted ‘balms’ based on relative peak areas, and, where present, corroborated by internal standards added at the extraction stage. Compositions do not imply that they were the original formulations due to possible chemical changes over time.

*after Brunton 1937.

The major products seen in all burials are degraded acyl lipids, derived from plant oils and animal fats (Table 1). Although some may derive from endogenous body lipids, most display non-human fatty acid distributions even after accounting for processes of decay. Both animal sterols, based on cholesterol, and plant sterols such as β-sitosterol were identified. The presence of plant oils and animal fats suggests that they were ingredients in a treatment involving the application of impregnated linen wrappings to these bodies. Indeed the proportion of animal fat/plant oil in the extracted ‘resin’ impregnating the textiles ranges from 64–95%, with a mean of 76% (RSD = 11.5%), which compares with a mean of 74% fat/oil observed in previous studies [13].

Assigning a more precise plant or animal origin based purely on the fatty acids present is extremely challenging, and indeed often not possible, due to degradation of major unsaturated components, as evidenced by the presence of short chain dicarboxylic acids, and mono- and dihydroxycarboxylic acids indicative of autoxidation. However, although necessarily tentative, and further chemical investigations would be necessary, it is highly notable that in the sample extracts from all the Badarian period burials, i.e. 33.30.44, 33.30.72, 33.30.80 and 33.30.83, unusual 2-hydroxy (C_22_–C_26_) and 2,3-dihydroxy (C_24_–C_26_) long chain fatty acids, along with phytanic acid and 3-hydroxy (C_13_–C_18_) fatty acids (usually bacterially-derived), are significant components, which can be indicative of a marine invertebrate oil [29], and are notably typical of *Porifera* (sponges) [30].

Interestingly, in burials 33.30.80 and 33.30.83 several 5,6-secosteroids were also tentatively identified, although their precise structures have yet to be determined. Along with 5-oxo-5,6-secocholest-3-en-6-oic acid, a 5,6-secocholest-7-en-3,5,6-triol isomer (tentatively identified based on relative retention time and its mass spectrum) is consistent with the triol, hipposterol (5,6-secocholest-7-en-3β,5β,6-triol), and is present as a significant component of the extracts, along with two other 5,6-secosteroids, very tentatively identified as 5,6-secocholesta-3,6/7-dien-5-one and 5-oxo-5,6-secocholesta-3,7-dien-6-ol (Figures S18, S20–S23 in Appendix S1). Although more work is undoubtedly required, it is perhaps notable that hipposterol specifically (albeit only tentatively identified here) has been identified in the Mediterranean sponge *Hippospongia communis* [31], and not only would other 5,6-secosterols, such as those observed in this study, be expected degradation products of hipposterol, but they have also been identified in the lipids of *Hippospongia communis* [32].

Secosteroids and secosterols of marine invertebrate origin, specifically sponges, are well known and have been the subject of a number of studies [33], [34], [35], [36], [37]. These secosteroids, specifically 5,6-secosteroids, can theoretically result from the oxidation of cholesterol, but although the C5–C6 double bond in cholesterol can be easily oxidised, the secosterols observed here are not easily formed. To produce the 5,6-secocholesterols observed, either the highly oxidising ozone (O_3_) or singlet oxygen via Hock-cleavage is normally necessary [38], [39], [40]. Where C-C bond scission does occur, the 5,6-secosterol without subsequent cyclization, i.e. the components observed in these archaeological samples, is either not formed at all, or is only formed as a very minor component, with the recyclized analogue being by far the major component formed in the human body, for example [38], [40], yet notably absent in these archaeological samples.

Moreover, the presence of phytanic acid is also intriguing. It can be indicative of a ruminant/dairy source, but also occurs in marine organisms [41]. In many marine animals it is often accompanied by the isoprenoid 4,8,12-trimethyltridecanoic acid [42], reflecting dietary intake. In this context it is notable that all Badarian samples containing phytanic acid have an absence of its isoprenoid counterpart, 4,8,12-trimethyltridecanoic acid; this is, perhaps notably, consistent with a sponge source where the organism preferentially produces only one of these isoprenoid acids, to the exclusion of the other [43].

Taken together these highly unusual fatty acids and steroidal compounds provide possible evidence of a marine invertebrate source, possibly sponge, in the Badarian period burials. In marked contrast, these compounds were only detected in one of the samples investigated from the succeeding Predynastic (Naqada IIB-C) burials, and in this sample they were only a relatively small constituent of the recipe (2% compared to up to 22% in the Badarian samples). Whether this change reflects greater difficulty in accessing resources, or less reliance on and need for marine products, is not yet clear. However, with the change from seasonally mobile pastoralism during the Late Neolithic (Badarian) period with known connections to the Red Sea, to an increasing dependence on agriculture and a more sedentary existence along the Nile Valley in the Predynastic period, it is perhaps significant that this transition occurs around 3800 – 3600 BC from the latest research [20], which agrees well with the radiocarbon dating on these Badarian and Predynastic burials (Fig. 1). In this context, it is perhaps interesting to note that around the time of this Badarian/Predynastic transition, scientific studies have shown that there were significant and relatively sudden changes in climate in the eastern Mediterranean, including parts of Egypt and the northern Red Sea [44], [45].

Plant wax was identified in several of the samples (Table 1) and the same chemical profile observed in all, regardless of whether matting, linen or ‘resin’. The same suite of wax-derived compounds were observed in both the Badarian and Naqada period burials pointing to the same natural source forming part of the recipes applied to the bodies as part of the funerary process. The plant wax constituents detected included C_12_, C_16_, C_18_, C_22_, C_24_, C_26_ and C_28_ *n*-alkanols, C_22:0_ to C_34:0_ long chain fatty acids with a marked even over odd predominance, and the C_28_ and C_40_ *n*-alkanes. Although the *n*-alkanols, long chain fatty acids and *n*-alkanes are well known to be major constituents in plant waxes [46], [47] their widespread natural occurrence makes identifying a likely specific source a significant challenge in the absence of possible candidates, and consequently it is beyond the remit of this current study.

**Figure 3:**
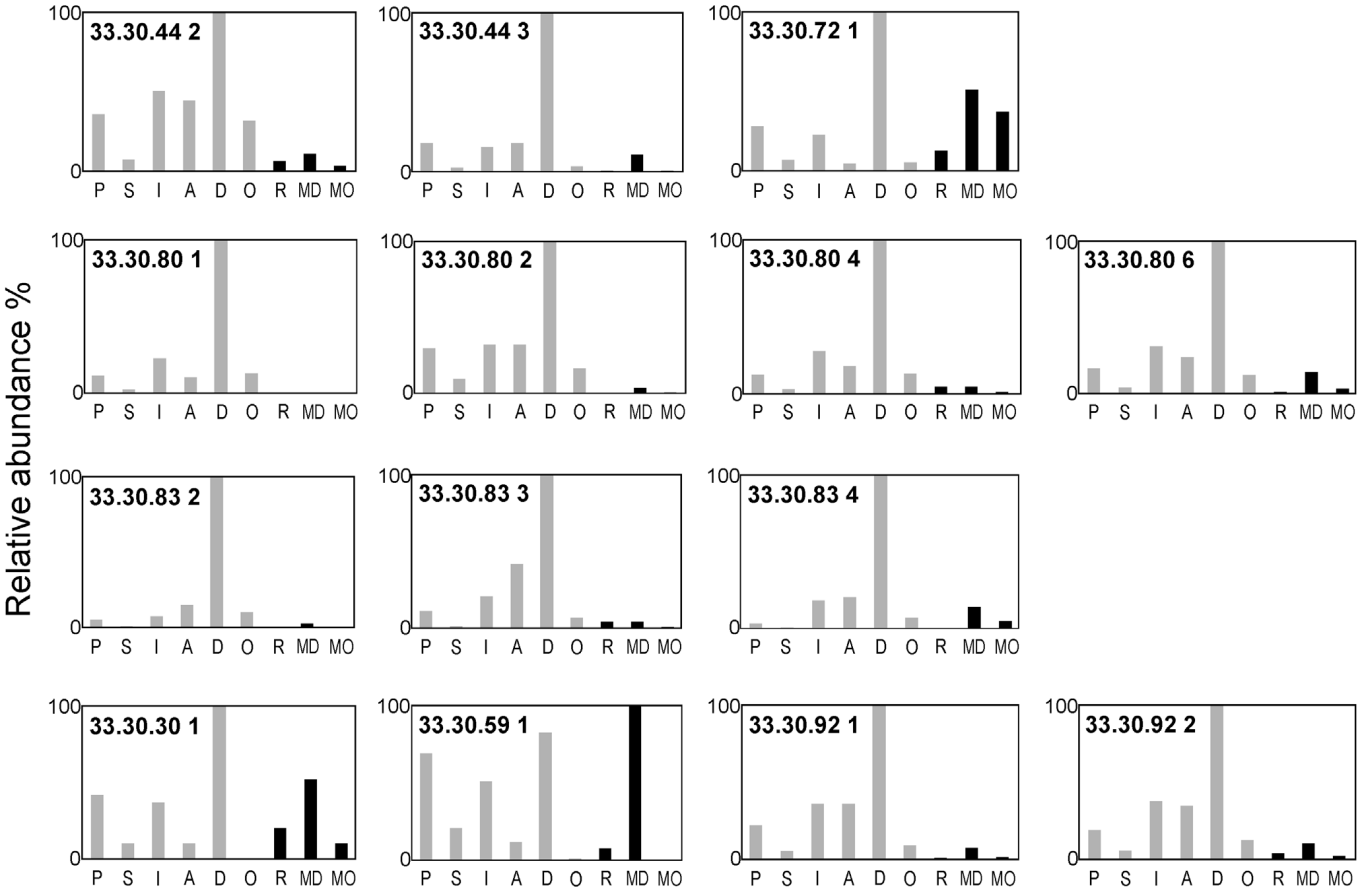
Chemical analyses of samples taken from the bodies and their wrappings in this research. Histograms show the distributions of diterpenoid acids: P = pimaric acid, S = sandaracopimaric acid, I = isopimaric acid, A = abietic acid, D = dehydroabietic acid, O = 7-oxodehydroabietic acid, R = retene, MD = methyl dehydroabietate, MO = methyl 7-oxodehydroabietate present in samples where pine resin has been specifically identified (see Table 1). The retene, methyl dehydroabietate and methyl 7-oxodehydroabietate reflect the degree of heating/processing the pine resin has undergone, prior to application to the linen/bodies as part of the ancient recipes.

Coniferous resin was present in all samples analyzed (Table 1) with both functionalized and defunctionalized diterpenoid compounds observed. Those functionalized resin acids identified included pimaric acid, sandaracopimaric acid, isopimaric acid, 6-dehydrodehydroabietic acid, dehydroabietic acid, abietic acid, 15-dehydrodehydroabietic acid, 6-hydroxydehydroabietic acid, 7-hydroxydehydroabietic acid, 15-hydroxydehydroabietic acid, 7-oxodehydroabietic acid and 15-hydroxy-7-oxodehydroabietic acid, these last five oxidized species, particularly 7-oxodehydroabietic acid, typical of aged archaeological conifer resins [6], [10], [12], [13], [48]. Dehydroabietic acid and 7-oxodehydroabietic acid were usually the major diterpenoid components (Fig. 3), which is notably similar to the dominant coniferous biomarkers observed in previous studies on ‘balms’ in Pharaonic and Graeco-Roman mummies [10], [12], [13].

Also identified in the conifer resin of all burials studied was the defunctionalized diterpenoid retene, methyl dehydroabietate and methyl 7-oxodehydroabietate, with minor amounts of the 19- and 18-norabieta-8,11,13-trienes. In high abundance these three compounds (and the norabietatrienes) are normally regarded as evidence for a conifer ‘pitch’ or ‘tar’ following strong heating [49], [50], [51]. In contrast, these ‘pitch markers’ are often absent, or present in very low abundance (retene ∼1%, methyl dehydroabietate ∼2%, methyl 7-oxodehydroabietate trace or absent), in a fresh unheated conifer resin [51]. In true conifer ‘pitches’ or ‘tars’ their abundance is far higher with typically ∼10–30% retene and ∼20–40% methyl dehydroabietate [49], [50], [51]. The samples in this study contained an average of 2.1% retene, 10.2% methyl dehydroabietate and 1.7% methyl 7-oxodehydroabietate as a proportion of the total coniferous resin diterpenoids (Fig. 3). Therefore, the presence of retene and these resin acid methyl esters in moderate abundance unequivocally identify purposeful processing of this ingredient of the ‘balms’, beyond simple physical mixing.

**Figure 4:**
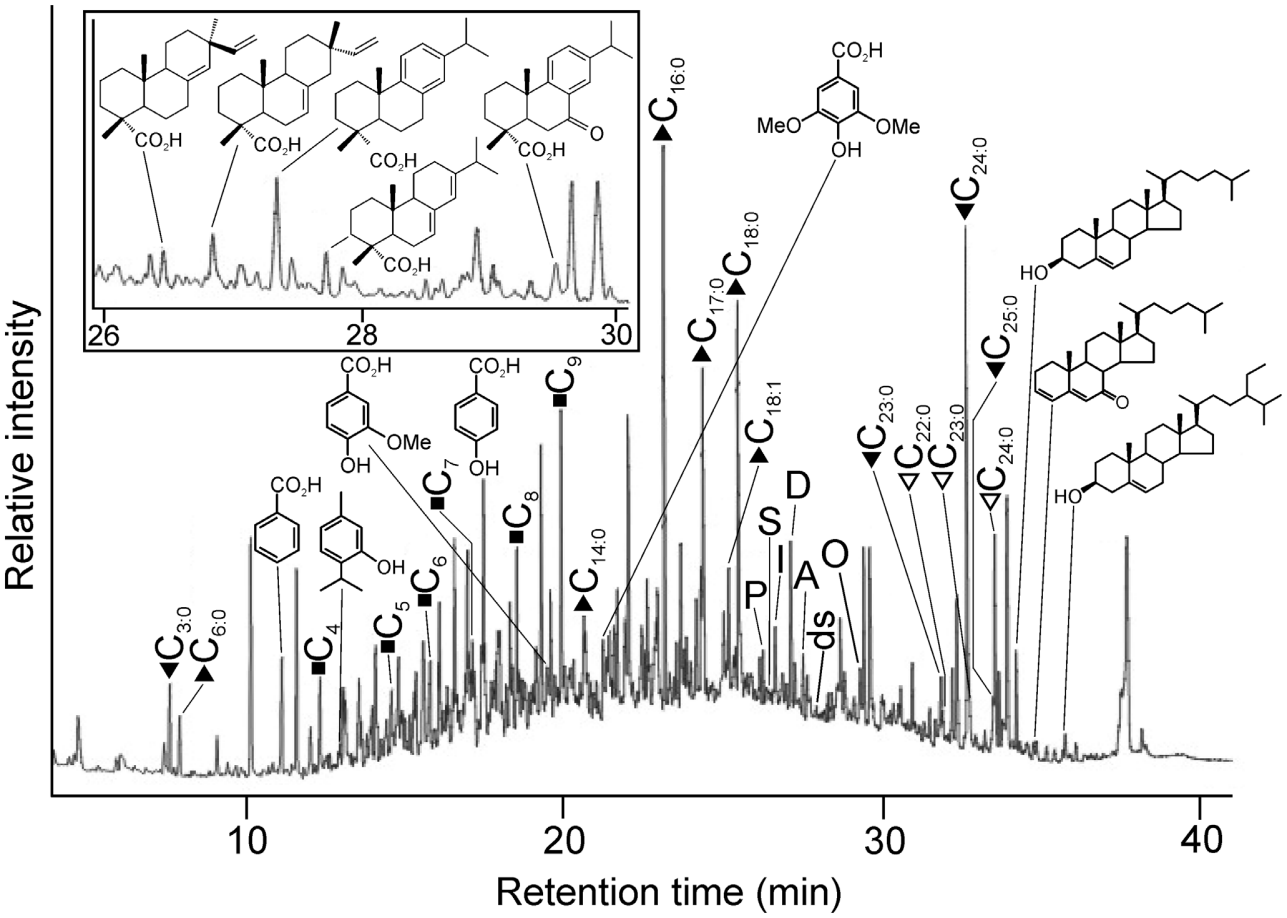
Reconstructed gas chromatography-mass spectrometry (GC-MS) total ion chromatogram (TIC) of the trimethylsilylated total lipid extract of 33.30.44 2. Peak identities (‘n’ indicates carbon chain length; where shown, i indicates degree of unsaturation): filled triangles, C_n:i_ indicates fatty acids; filled squares, C_n_ indicates α,ù-dicarboxylic acids; filled inverted triangles, C_n:i_ indicates 2-hydroxy fatty acids; open inverted triangle, C_n:i_ indicates 2,3-dihydroxy fatty acid. Also shown are the structures of four aromatic acids identified: benzoic acid, 4-hydroxybenzoic acid, vanillic acid (4-hydroxy-3-methoxybenzoic acid) and syringic acid (4-hydroxy-3,5-dimethoxybenzoic acid); one monoterpenoid: thymol; six diterpenoids: pimaric acid (P), sandaracopimaric acid (S), isopimaric acid (I), dehydroabietic acid (D), abietic acid (A) and 7-oxo-dehydroabietic acid (O) (labeled as in Figure 3) and four steroidal compounds identified: stigmasta-3,5,22-triene, cholesterol, cholesta-3,5-dien-7-one and β-sitosterol; the letters ds represent a disaccharide. Inset displays a partial reconstructed GC-MS TIC of this sample focusing on the diterpenoid (resin) acids and showing the molecular structures of five of those identified: pimaric acid, isopimaric acid, dehydroabietic acid, abietic acid and 7-oxodehydroabietic acid.

The presence of pimaric and sandaracopimaric acid in a ratio of >2∶1 (average ∼4.5∶1; Fig. 3) identifies a temperate *Pinus* species, i.e. pine, as the source of the resin in those samples [52]. Proportions of 0.1–11% conifer resin components in the ‘resin’ applied to the textiles (Figs. 3 and 4 (inset)) is consistent with the typical proportions of conifer resin measured in Pharaonic mummies (0.2–6%) [13]. The use of coniferous resins is most notable since their terpenoids are known to inhibit microbial degradation via mechanisms (physico-chemical barriers and antimicrobial action) analogous to their protective roles in the plants from which they were derived [53].

Significant compounds in many of the samples were aromatic acids characteristic of plant products. Their profiles and wide distribution in nature make the precise identification of the source extremely difficult pending further investigation. The possibility that they derive from the *Juncus* or Halfa reed matting that covered these wrapped bodies cannot be ruled out entirely, though their derivation from reed-based lignin is perhaps unlikely, due to the absence of the major lignin building-block compounds (*p*-hydroxyphenyl-, guaiacyl and syringyl moieties) in any of the Py-GC-MS analyses [54]. Moreover, the relative distributions of the aromatic acids present in the linen wrappings is not consistent with the expected degradation products of lignin from a flax source [55], or monocots such as *Juncus* or Halfa [56].

Present in all burials and samples analyzed, the aromatic acids in the Badarian and later Predynastic (Naqada IIB-C) are dominated by 4-hydroxybenzoic acid or syringic acid (4-hydroxy-3,5-dimethoxybenzoic acid). Pharaonic percentages of ‘balsam’/aromatic plant extracts in balms (trace-16% [13]) are comparable to those in the Badarian and Predynastic/Naqada period textile, ‘resin’ and skin samples (2.4–20%; Table 1). The higher contribution of the aromatic plant extracts to the ‘balms’ of those samples comprising reed matting (24–54%), combined with a somewhat different chemical composition of the particular aromatic acids present in these samples, may suggests an alternative source, or sources, although the chemical profiles are inconsistent with the expected lignin degradation products from the reed matting [56].

**Figure 5:**
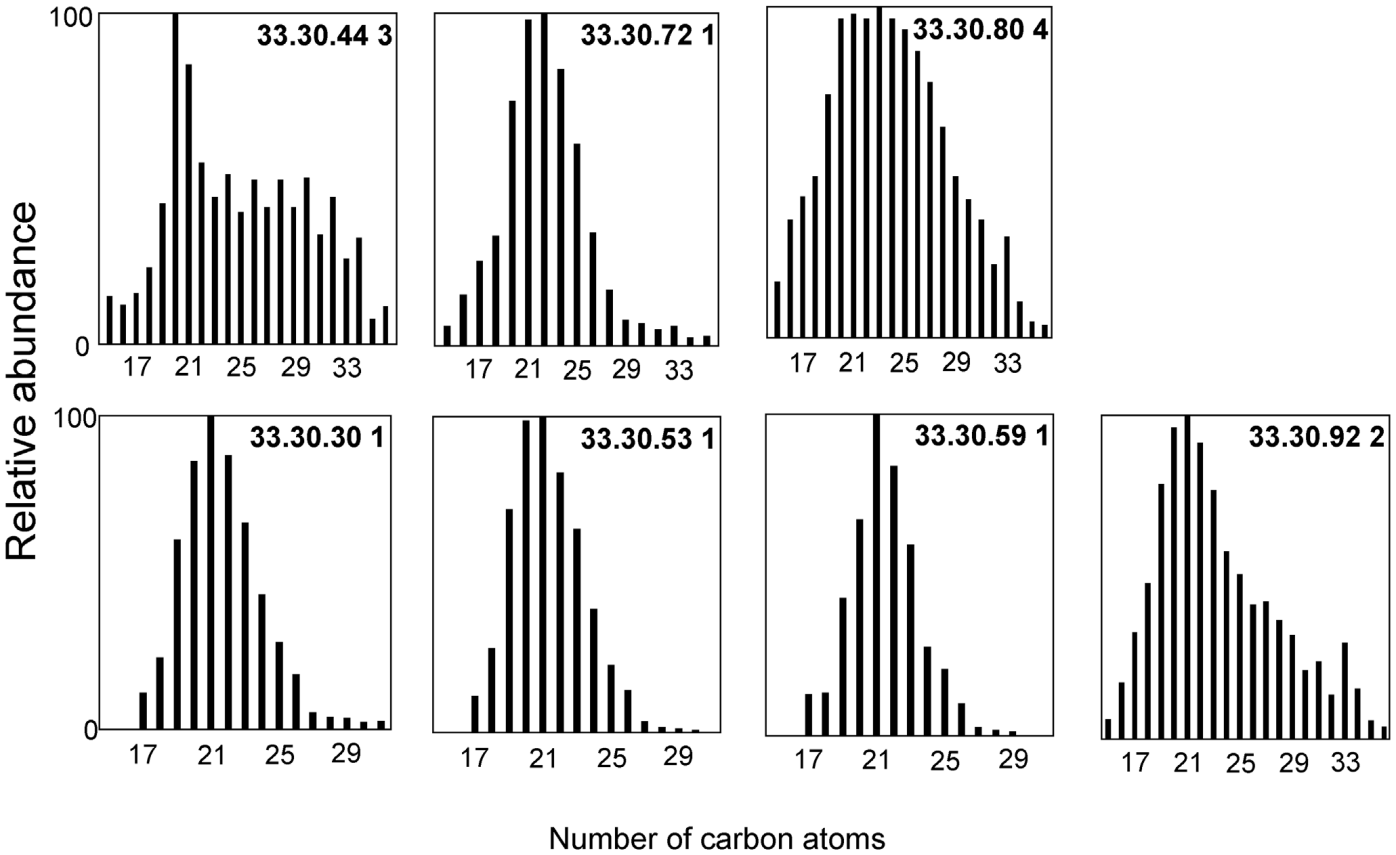
Chemical analyses of samples taken from the bodies and their wrappings in this research. Histograms show the distributions of C_15+_ n-alkanes from samples containing a natural petroleum seep (the samples containing the highest abundance of natural petroleum for each burial are presented; see Table 1).

**Figure 6:**
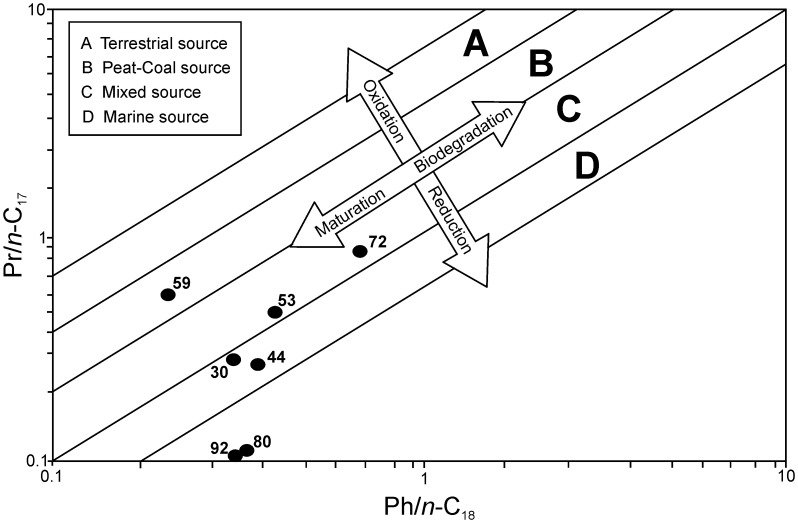
Plot of Pr/*n*-C_17_ versus Ph/*n*-C_18_ cross plot showing the source and depositional environments of the natural petroleum seeps (the samples containing the highest abundance of natural petroleum for each burial are presented; see **Table 1**): 44 = 33.30.44 3, 72 = 33.30.72 1, 33.30.80 4, 30 = 33.30.30 1, 53 = 33.30.53 1, 59 = 33.30.59 1, 92 = 33.30.92 2.

A natural petroleum hydrocarbon source was also present in most samples (Table 1), evidenced by the presence of a homologous series of *n*-alkanes (∼C_10_–C_36_) in the GC-MS analyses. Notably, these paraffinic profiles very clearly point to different natural sources between burials (Fig. 5). Plots of Pr/*n*-C_17_ vs Ph/*n*-C_18_ (Fig. 6) point to a marine source for samples from burials 33.30.44, 33.30.80, 33.30.30 and 33.30.92 [57], further corroborated by the abundance of C_11_–C_22_ *n*-alkanes in all but burial 33.30.30, which are indicative of marine microorganisms and algae/phytoplankton [58], [59], [60]. Burial 33.30.44 displays a bimodal distribution of *n*-alkanes with a slight even over odd predominance (carbon preferential index, CPI = 0.78) and maximising at C_20_, which may suggest a greater input from marine microorganisms in addition to marine algae/phytoplankton [58], [59], [60]. The notably bimodal distributions of 33.30.80 (maximising at C_23_ and C_33_) and 33.30.92 (maximising at C_21_ and C_33_) also indicates a small terrestrial input into both these natural petroleum sources. Although essentially mono-modal (maximising at C_21_), 33.30.30 shows a slight odd over even predominance from C_27_–C_33_ (CPI = 1.12) indicating a very small terrestrial input in the natural petroleum. Burials 33.30.72 and 33.30.53 display a mono-modal distribution of *n*-alkanes with no significant odd over even predominance and maximising at C_21_. A plot of Pr/*n*-C_17_ vs Ph/*n*-C_18_ points to a mixed source for these samples [57], with no C_11_–C_14_ *n*-alkanes. Burial 33.30.59 displays a mono-modal distribution of *n*-alkanes with no significant odd over even predominance and maximising at C_21_. A plot of Pr/*n*-C_17_ vs Ph/*n*-C_18_ suggests a peat-coal source for this sample [57].

From the analysis of the Py-GC-MS data (Figure S29 in Appendix S1) a thermally-derived component indicative of kerogen was present in all burials. The proportions of the aliphatic, naphthenic and aromatic components in the kerogen components of all burials were notably similar, ranging from 24–28% aliphatic, 0–1% naphthenic and 71–76% aromatic, suggesting a Type IIc kerogen in all cases [61], [62]. The possibility that the kerogen could derive from other components of the ‘resin’ samples as ‘analytical artefacts’ was carefully considered and can be discounted.

Despite selectively monitoring for the presence of ions m/z 191 (triterpanes) and *m*/*z* 217 (steranes), usually associated with a natural petroleum source, and characteristic of a true natural bitumen [11], these biomarkers were not detected in either the GC-MS or TD-GC-MS analyses. Given the small component this natural petroleum source constitutes of the ancient ‘resins’ it may be that the triterpanes and steranes are present in such low abundance they are below the detection limits of the current study and further investigations may prove more successful. In considering this, it may also reflect the absence of, or relatively small input from, bacteria, from whose cell walls these biomolecules derive [63]. Although seen as ubiquitous [63], steranes and triterpanes are absent in algae and non-photosynthetic bacteria [64], which would certainly be consistent with the biomarker data for the natural petroleum source from 33.30.44, 33.30.80, 33.30.30 and 33.30.92.

Consequently, the natural petroleum sources characterized in this study all consisted very largely of *n*-alkanes (Fig. 5). Chemically very similar paraffinic hydrocarbon oil seeps which are dominated by *n*-alkanes, with other components constituting only a very small amount of the natural petroleum source, are known [65]. Unfortunately, we are not aware of any specific sources in Egypt or the Near East, which may reflect their anciently highly symbolic, yet modern non-commercial, nature. In this context, the findings here are perhaps not unexpected and further research in this particular area is undoubtedly required. It should perhaps also be noted that although not directly associated with human remains, natural petroleum sources were known to have been exploited in Egypt during the 4^th^ millennium BC (Naqada I-Early Naqada II, c.3800 – 3500 BC), i.e. comparable with the samples analysed in this study [66].

The chemical evidence for monosaccharides, 6-deoxyhexoses, inositols, sugar acids (e.g. galacturonic acid) and disaccharides in the solvent extracts of samples analyzed suggest the possibility of a plant gum or sugar being a component of the ‘resin’, although the precise sources have yet to be identified. The presence of both pyranose (e.g. glucose) and furanose (e.g. fructose) moieties in the samples, in addition to characteristic distributions of these sugar markers within samples from the same burials, yet markedly different distributions between burials, argues against these components deriving, certainly wholly, from degraded cellulose-based linen [67]. The same rationale also excludes the possible degradation of the cellulose acetate (see Appendix S1) to explain the presence of these constituents. Moreover, the sugar/gum component was observed in similarly small proportions (trace-4%, with an average of 1%) in all samples, regardless of whether linen, ‘resin’, skin or matting. This together with the same ‘gum’ profile within burials from both cellulosic and non-cellulosic material giving the same ‘chemical fingerprint’ is strongly indicative of a plant gum/sugar. Notably, plant gum/sugar has previously been identified in Egyptian mummies [13], [67], [68] and the Greek historian and putative ‘Father of History’ Herodotus specifically mentions their use in mummification to secure the wrappings some 3000 years after their use in these prehistoric burials [69].

Our results reveal the utilization of fairly consistent compositions both in terms of constituents and the relative proportions of those ingredients, and also clear evidence for processing [50], [51]. More specifically, these recipes consist of a plant oil or animal fat ‘base’ constituting the bulk of the ‘balms’, with far lesser amounts of a conifer resin and an aromatic plant extract/‘balsam’, and minor amounts of a wax and a plant gum/sugar. These relative abundances are typical of those used in mummification throughout much of ancient Egypt's 3000 year Pharaonic history. Moreover, these recipes contained antibacterial agents, used in the same proportions as were employed by the Egyptian embalmers when their skill was at its peak, some 2500–3000 years later [13]. Both the antibacterial properties of some of the ingredients and the similarity of the recipes to those embalming agents utilized at the height of body preservation in ancient Egypt strongly suggest that these ‘resin’-impregnated textiles and the localized soft-tissue preservation they would have afforded, are the true antecedents of Egyptian mummification, practiced in some form for 5000 years. As such, the scientific findings presented here push the origins of this central and vital facet of ancient Egyptian culture back by some 1500 years.

## Supporting Information

Appendix S1 **Methods and detailed results: Microscopy, AMS radiocarbon dating and Chemical Analyses.**
(PDF)

## References

[1] Jones J (2007) New perspectives on the development of mummification and funerary practices during the Pre- and Early Dynastic Periods. Proceedings of Ninth International Congress of Egyptologists. Grenoble, 6–12 September 2004, Goyon J-C, Cardin C, Garrel J-F, Zaki, G, editors (Peeters, Leuven). pp 982–989.

[2] Ikram S, Dodson A (1998) The Mummy in Ancient Egypt: Equipping the Dead for Eternity. London: Thames and Hudson. p. 108.

[3] Aufderheide AC (2003) The Scientific Study of Mummies. Cambridge: Cambridge University Press. p. 253.

[4] David AR (2000) Mummification. In: Nicholson PT, Shaw I, editors. Ancient Egyptian Materials and Technology. Cambridge: Cambridge University Press. p. 373.

[5] Serpico M (2000) Resins, amber and bitumen. In: Nicholson PT, Shaw I, editors. Ancient Egyptian Materials and Technology. Cambridge: Cambridge University Press. p. 465.

[6] Colombini MP, Modugno F, Silvano F, Onor M (2000) Characterization of the balm of an Egyptian mummy from the seventh century B.C. Studies in Conservation 45 (1) 19–29.

[7] Connan J (1991) Le bitume des momies égyptiennes, un passport pour l'éternité (French: Bitumen in Egyptian mummies, a passport to eternity). Recherche 238: 1503–1504.

[8] Harrell JA, Lewan MD (2002) Sources of mummy bitumen in ancient Egypt and Palestine. Archaeometry 44 (2) 285–293.

[9] Maurer J, Moehring T, Rullkötter J, Nissenbaum A (2002) Plant lipids and fossil hydrocarbons in embalming material of Roman Period mummies from the Dakhleh Oasis, Western Desert, Egypt. J Archaeol Sci 29: 751–762.

[10] Proefke ML, Rinehart KL, Raheel M, Ambrose SH, Wisseman SU (1992) Probing the mysteries of ancient Egypt: chemical analysis of a Roman Period Egyptian mummy. Anal Chem 64 (2) 105A–111A.

[11] Rullkötter J (1988) Nissenbaum (1988) A Dead Sea asphalt in Egyptian mummies: molecular evidence. Naturwissenschaften 75: 618–621. 3237249 10.1007/BF00366476

[12] Koller J, Baumer U, Kaup Y, Etspüler H, Weser U (1998) Embalming was used in Old Kingdom. Nature 391: 343–344. 9450745 10.1038/34809

[13] Buckley SA, Evershed RP (2001) Organic chemistry of embalming agents in Pharaonic and Graeco-Roman mummies. Nature 413 (6858) 837–841. 11677605 10.1038/35101588

[14] Buckley SA, Stott AW, Evershed RP (1999) Studies of organic residues from ancient Egyptian mummies using high temperature-gas chromatography-mass spectrometry and sequential thermal desorption-gas chromatography-mass spectrometry and pyrolysis-gas chromatography-mass spectrometry. Analyst 124: 443–452. 10605875 10.1039/a809022j

[15] Koller J, Baumer U, Kaup Y, Schmid M, Weser U (2003) Analysis of a pharaonic embalming tar. Nature 425: 784. 14574400 10.1038/425784a

[16] Hendrickx S (2006) In: Hornung E, Krauss R, Warburton DA editors. Ancient Egyptian Chronology. Handbook of Oriental Studies. Leiden/Boston: Brill. pp. 55–93.

[17] Taylor JH (2001) Death and the Afterlife in Ancient Egypt. London: The British Museum Press. p. 48.

[18] Brunton G, Caton Thompson G (1928) The Badarian Civilisation and Prehistoric Remains near Badari. London: Bernard Quaritch. p. 19.

[19] Brunton G (1937) Mostagedda and the Tasian Culture. London: Bernard Quaritch.

[20] Dee M, Wengrow D, Shortland A, Stevenson A, Brock F, et al. (2013) An absolute chronology for early Egypt using radiocarbon dating and Bayesian statistical modelling. Proc R Soc A 469: 20130395 - 4^th^ September 2013 (doi:10.1098/rspa.2013.0395) 10.1098/rspa.2013.0395 PMC3780825 24204188

[21] Friedman R, Wattrall E, Jones J, Fahmy AG, Van Neer W, et al (2002) Excavations at Hierakonpolis. Archéo-Nil 12: 55–68.

[22] Hendrickx S, Van Den Brink ECM (2002) In: Levy TE, Van Den Brink E, editors. Egypt and the Levant. Interrelations from the 4^th^ through the Early 3^rd^ Millenium BCE. London/New York: Leicester University Press. pp. 346–398.

[23] Jones J (2008) Pre- and Early Dynastic Textiles. Technology, specialisation and administration during the process of State formation. In: Midant-Reynes B, Tristant Y, editors, with the collaboration of Joanne Rowland and Stan Hendrickx. Egypt at its Origins 2. Proceedings of the International Conference ‘Origin of the State. Predynastic and Early Dynastic Egypt’, Toulouse, 5^th^ to 8^th^ September, 2005. Leuven: Peeters. pp. 99–132.

[24] Rost FWD, Oldfield RJ (2000) Photography with a Microscope. Cambridge: Cambridge University Press. p. 288.

[25] Oldfield RJ (1994) Light Microscopy: an Illustrated Guide. London: Wolfe Publishing. p. 160.

[26] Dee M, Bronk Ramsey C (2000) Refinement of Graphite Target Production at ORAU. Nucl Instrum Meth Phys Res B 172: 449–453.

[27] Bronk Ramsey C (2009) Bayesian analysis of radiocarbon dates. Radiocarbon 51: 337–360.

[28] Stuiver M, Polach HA (1977) Discussion: Reporting of ^14^C Data. Radiocarbon 19: 355–363.

[29] Faulkner DJ (1994) Marine Natural Products. Nat Prod Rep 11: 355–394. 15200019 10.1039/np9941100355

[30] Barnathan G, Kornprobst J-M, Doumenq P, Miralles J, Boury-Esnault N (1993) Sponge fatty acids, 5. Characterization of complete series of 2-hydroxy long-chain fatty acids in phospholipids of two Senegalese marine sponges from the family *Suberitidae*: *Pseudo*s*uberites* and *Suberites massa* . J Nat Prod 56 (12) 2104–2113.

[31] Madaio A, Piccialli V, Sica D (1998) Hipposterol, a unique trihydroxylated 5,6-secosterol from the marine sponge *Hippospongia communis* . Tetrahedron Lett 29 (46) 5999–6000.

[32] Madaio A, Notaro G, Piccialli V, Sica D (1990) Minor 5,6-secosterols from the marine sponge *Hippospongia communis*. Isolation and synthesis of (7*Z*,22*E*,24*R*)-24-methyl-5,6-secocholesta-7,22-diene-3β,5β,6-triol. J Nat Prod 53 (3) 565–572.

[33] Sica D, Musumeci D (2004) Secosteroids of marine origin. Steroids 69: 743–756. 15579326 10.1016/j.steroids.2004.09.001

[34] Dopeso J, Quiñoá E, Riguera R, Debitus C, Bergquist PR (1994) Euryspongiols: Ten New Highly Hydroxylated 9,11-Secosteroids with Antihistaminic Activity from the Sponge *Euryspongia* sp. Stereochemistry and Reduction. Tetrahedron 50 (12) 3813–3828.

[35] Bonini C, Cooper CB, Kazlauskas R, Wells RJ, Djerassi C (1983) Minor and Trace Sterols in Marine Invertebrates. 41. Structure and Stereochemistry of Naturally Occurring 9,11-Seco Sterols. J Org Chem 48 (12) 2108–2111.

[36] Aiello A, Esposito G, Fattorusso E, Iuvone T, Luciano P, et al (2003) Aplidiasterols A and B, two new cytotoxic 9,11-secosterols from the Mediterranean ascidian *Aplidium conicum* . Steroids 68: 719–723. 14625003 10.1016/s0039-128x(03)00098-9

[37] Laurent D, Pietra F (2004) Natural-Product Diversity of the New Caledonian Marine Ecosystem Compared to Other Ecosystems: A Pharmacologically Oriented View. Chemistry and Biodiversity 1: 539–594. 17191868 10.1002/cbdv.200490048

[38] Wentworth AD, Songa B-D, Nievab J, Shaftona A, Tripurenania S, et al (2009) The ratio of cholesterol 5,6-*seco*sterols formed from ozone and singlet oxygen offers insight into the oxidation of cholesterol *in vivo* . Chem Commun 21: 3098–3100. 10.1039/b821584g PMC2819220 19462099

[39] Pulfer MK, Murphy RC (2004) Formation of Biologically Active Oxysterols during Ozonolysis of Cholesterol Present in Lung Surfactant. J Biol Chem 279 (25) 26331–26338. 15096493 10.1074/jbc.M403581200

[40] Tomono S, Miyoshi N, Sato K, Ohba Y, Ohshima H (2009) Formation of cholesterol ozonolysis products through an ozone-free mechanism mediated by the myeloperoxidase–H2O2–chloride system. Biochem Biophys Res Commun 383: 222–227. 19345674 10.1016/j.bbrc.2009.03.155

[41] Craig OE, Steele VJ, Fischer A, Hartz S, Andersen SH, et al (2011) Ancient lipids reveal continuity in culinary practices across the transition to agriculture in Northern Europe. Proc Natl Acad Sci USA 108 (44) 17910–17915. 22025697 10.1073/pnas.1107202108 PMC3207664

[42] Ackman RG, Hooper SN (1968) Examination of isoprenoid fatty acids as distinguishing characteristics of specific marine oils with particular reference to whale oils. Comp Biochem Physiol 24: 549–565. 5651292 10.1016/0010-406x(68)91008-6

[43] Carballeira NM, Maldonado L, Porras B (1987) Isoprenoid Fatty Acids From Marine Sponges. Are sponges selective? Lipids 22 (10) 767–769. 3431350 10.1007/BF02533980

[44] Hassan FA, Barich BA, Mahmoud M, Hemdan MA (2001) Holocene playa deposits of Farafra Oasis, Egypt, and their Palaeoclimatic and Geoarchaeological significance. Geoarchaeology 16 (1) 29–46.

[45] Robinson SA, Black S, Sellwood BW, Valdes PJ (2006) A review of palaeoclimates and palaeoenvironments in the Levant and Eastern Mediterranean from 25,000 to 5000 years BP: setting the environmental background for the evolution of human civilisation. Quaternary Science Reviews 25: 1517–1541.

[46] Tulloch AP (1973) Comparisons of Some Commercial Waxes by Gas Liquid Chromatography. J Am Oil Chem Soc 50 (9) 367–371.

[47] Tulloch AP (1974) Composition of some natural waxes. Cosmetics and Perfumery 89 (11) 53–54.

[48] Pollard AM, Heron C (1996) Archaeological Chemistry. Cambridge: The Royal Society of Chemistry. p. 247.

[49] Robinson N, Evershed RP, Higgs WJ, Jerman K, Eglinton G (1987) Proof of a Pine Wood Origin for Pitch from Tudor (Mary Rose) and Etruscan Shipwrecks: Application of Analytical Organic Chemistry in Archaeology. Analyst 112: 637–644.

[50] Beck CW, Stout EC, Bingham J, Lucas J, Purohit V (1999) Central European pine tar technologies. Ancient Biomolecules 2 (4) 281–293.

[51] Hjulström B, Isaksson S, Hennius A (2006) Organic geochemical evidence for pine tar production in middle Eastern Sweden during the Roman Iron Age. J Archaeol Sci 33: 283–294.

[52] van der Werf ID, van den Berg KJ, Schmitt S, Boon JJ (2000) Molecular characterization of copaiba balsam as used in painting techniques and restoration procedures. Studies in Conservation 45: 1–18.

[53] Briggs DEG, Eglinton G (1994) Chemical traces of ancient life. Chemistry in Britain 30: 907–912.

[54] Boerjan W, Ralph J, Baucher M (2003) Lignin Biosynthesis. Annu Rev Plant Biol 54: 519–546. 14503002 10.1146/annurev.arplant.54.031902.134938

[55] del Río JC, Rencoret J, Gutiérrez A, Nieto L, Jiménez-Barbero J, et al (2011) Structural Characterization of Guaiacyl-rich Lignins in Flax (Linum usitatissimum) Fibers and Shives. J Agric Food Chem 59: 11088–11099. 21905657 10.1021/jf201222r

[56] Clifford DJ, Carson DM, McKinney DE, Bortiatynski JM, Hatcher PG (1995) A new rapid technique for the characterization of lignin in vascular plants: thermochemolysis with tetramethylammonium hydroxide (TMAH). Org Geochem 23 (2) 169–175.

[57] Younes MA, Philip RP (2005) Source Rock Characterization based on Biological Marker Distribution of Crude Oils in the Southern Gulf of Suez, Egypt. J Petrol Geol 28 (3) 301–317.

[58] Grimalt J, Albaiges J (1987) Sources and occurrence of C_12_–C_22_ n-alkane distributions with even carbon number preference in sedimentary environments. Geochim Cosmochim Acta 51: 1379–1384.

[59] Punyu VR, Harji RR, Bhosle NB, Sawant SS, Venkat K (2013) *n*-Alkanes in surficial sediments of Visakhapatnam harbour, east coast of India. J Earth Syst Sci 122 (2) 467–477.

[60] Elias VO, Cardoso JN, Simoneit BRT (2000) Acyclic Lipids in Amazon Shelf Waters. Estuar Coast Shelf Sci 50: 231–243.

[61] Vandenbroucke M (2003) Kerogen: from Types to Models of Chemical Structure. Oil Gas Sci Technol 58 (2) 243–269.

[62] Larter SR, Douglas AG (1980) A pyrolysis-gas chromatographic method for kerogen typing. Physics and Chemistry of the Earth 12: 579–583.

[63] Mills JS, White R (1994) The Organic Chemistry of Museum Objects. Oxford: Butterworth-Heinemann. pp. 56–58.

[64] Han J, Calvin M (1969) Hydrocarbon distribution of algae and bacteria, and microbiological activity in sediments. Proc Natl Acad Sci USA 64 (2) 436–443. 5261025 10.1073/pnas.64.2.436 PMC223361

[65] Clifton CG, Walters CC, Simoneit BRT (1990) Hydrothermal petroleums from Yellowstone National Park, Wyoming, USA. Appl Geochem 5: 169–191.

[66] Connan J, Nissenbaum A, Dessort D (1992) Molecular archaeology: Export of Dead Sea asphalt to Canaan and Egypt in the Chalcolithic and Bronze Age (4^th^-3^rd^ millennium BC). Geochim Cosmochim Acta 56: 2743–2759.

[67] Buckley SA, Clark KA, Evershed RP (2004) Complex Organic Chemical Balms of Pharaonic Animal Mummies. Nature 431 (7006) 294–299. 15372029 10.1038/nature02849

[68] Mejanelle P, Bleton J, Goursaud S, Tchapla A (1997) Identification of phenolic acids and inositols in balms and tissues from an Egyptian mummy. J Chromatogr A 767: 177–186. 9177008 10.1016/s0021-9673(96)01067-9

[69] Herodotus (c. 440 BC) Book II. In: Herodotus, The Histories. Trans. De Sélincourt A. (1954). Harmondsworth: Penguin. pp. 160–161.

