# Supplementary material for: Evidence for Prehistoric Origins of Egyptian Mummification in Late Neolithic Burials
Source: PLoS One. 2014 Aug 13;9(8):e103608. doi: 10.1371/journal.pone.0103608 (PMC4132097; doi:10.1371/journal.pone.0103608)
Supplement: Appendix S1 — Methods and detailed results: Microscopy, AMS radiocarbon dating and Chemical Analyses. (PDF) [file pone.0103608.s001.pdf]

Supporting Information: Appendix S1

Evidence for prehistoric origins of Egyptian mummification in Late Neolithic burials

Jana Jones[1], Thomas F.G. Higham[2], Ron Oldfield[3], Terry P. O'Connor[4], Stephen A. Buckley[4,5]

[1] *Department of Ancient History, Macquarie University, Sydney, New South Wales, Australia*
[2] *Oxford Radiocarbon Accelerator Unit, Research Laboratory for Archaeology and the History of Art, University of Oxford, Oxford, United Kingdom.*
[3] *Department of Biological Sciences, Macquarie University, Sydney, New South Wales, Australia*
[4] *Department of Archaeology, University of York, The Kings Manor, York, United Kingdom*
[5] *BioArch, Departments of Archaeology, Biology and Chemistry (S-Block), University of York, York, United Kingdom*

Author Information To whom correspondence should be addressed.
Email: sb55@york.ac.uk and jana.jones@mq.edu.au

Museum No.	Tomb No.	Sex	Condition*	Historical date	Characteristic objects	Reference
33.30.44	3538	F	Intact	Badarian	Bowl SB5h	Brunton 1937: 43, pl. 10
33.30.72	494	F	Intact	Badarian	Pot SB44p; clay figurine	Brunton 1937: 36, pls. 7, 14, 19, 24, 26; Ucko 1962: 19
33.30.80	1215	M	Disturbed	Badarian	Pot BR25e; rectangular palette	Brunton 1937: pls. 8,17, 23, 25
33.30.83	1214	F	Intact?	Badarian	Pot BB71d	Brunton 1937: pls. 8, 15, 19
33.30.30	11725	F?	Partly disturbed	Naqada IIB	Pots R81v; R93a; L12b	Brunton 1937: pls. 31, 36
33.30.53	1609	M	Almost intact	Naqada IIC	Pots 2 x R81; R94	Brunton 1937: pls. 29, 36
33.30.59	1640	M	Partly disturbed	Predynastic	No pottery	Brunton 1937: pl. 29
33.30.92	1637	F	Partly disturbed	Naqada IIC	Pots D8, B15, B21	Brunton 1937: pls. 29, 33, 35

*Condition based on Brunton 1937.

Table S1: Archaeological context of samples analysed. Sex of the occupant, condition of the tomb and grave goods were recorded in the excavation report. Only diagnostic objects are included here; beads, shells, flints, tools and bone objects have been omitted. The pottery types refer to W.M.F. Petrie's typological corpus [1].

Supplementary Methods

Microscopy

The presence of resin in and on some Badarian and Predynastic funerary wrappings at Mostagedda was hypothesized originally by Jana Jones. This observation was initially investigated by Oldfield and Jones using microscopical examination.

All the textile specimens from the Badarian and Predynastic period burials in the collection of the Bolton Library, Museum, and Archive Service were examined and documented, and the most significant were photographed. Altogether, 98 samples from Armant, Badari, Fayum, Gerzeh, Mostagedda and Matmar were taken for further microscopical analysis at Macquarie University, Sydney, Australia. Of these, nine samples of funerary wrappings from Badari and 42 from Mostagedda were examined microscopically for traces of 'resins'. Badarian and Predynastic samples from Mostagedda were selected for chemical investigation because they comprised the greatest quantity, and would provide a time series from that site.

Samples that presented themselves as likely to have 'resin' were photographed macroscopically and then through a high quality stereomicroscope, with incident twin-armed fiberoptic illumination [2].

The most convincing depiction of the 'toffee-like' (presumed) resin was effected in the light microscope using HD (Hellfeld/Dunkelfeld = Brightfield/Darkfield) objectives on a Zeiss Universal microscope [3]. The supposed resin revealed itself as a brownish substance on the outside of the cells. In microscopic specimens it was observed that the 'resin' had been incorporated into the cell walls. We were unable to dissolve the 'resin' in standard solvents used, but in some specimens there was a leaching of colour into the glycerine mountant after a period of 24 hours or more. The preservation of flax cells varied from superb to an opaque mass.

This darkfield epi-illumination technique requires very strong illumination but gives an image with brilliant contrast well suited to very high quality colour photography. There was no heat damage to the specimen, but the major disadvantage was the very limited and virtually uncontrollable depth of field. The specimen required very careful manipulation, occasionally on a cup-stage as used by gemmologists, so that it was oriented absolutely normal to the objective optical alignment.

Further investigation involved the excision of a piece of yarn some 3 to 4 mm in length from a less conspicuous part of the specimen. The yarn was placed in a drop of glycerine on a standard 3" x 1" microscope slide, carefully teased apart with a pair of mounted needles, and the preparation covered with a No 1 cover slip. Although the aim of the preparation was to display isolated fiber cells, the 'teasing apart' of the yarn needs to be done with some caution. In the preparation of the fiber for spinning into yarn there may

have been incomplete separation of the ultimates (flax fiber cells), and this feature should be preserved at least in parts of the specimen.

In the microscope with transmitted light illumination, we found darkfield, phase contrast and differential interference contrast techniques to be minimally useful. The most revealing information was by rapid comparison of brightfield and polarisation (crossed polars) images. A rotating stage was essential to the technique. The sheer beauty of polarisation images had much to commend it.

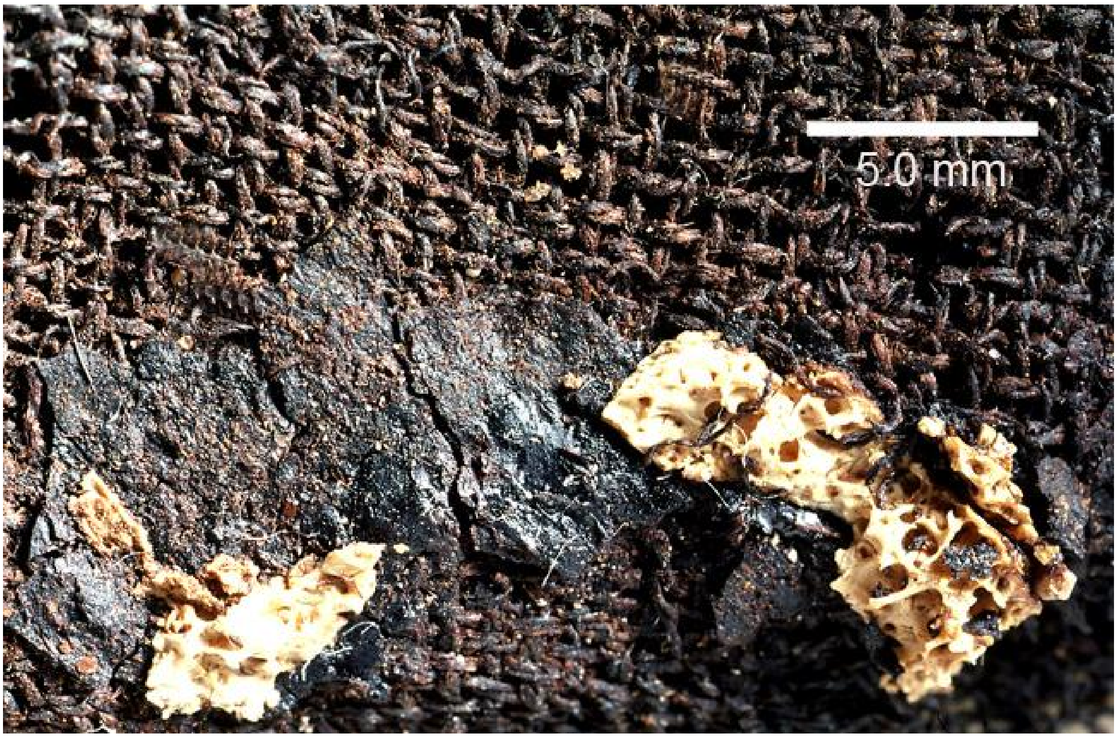

Figure S1: 33.30.20 Tomb No. 11742, Predynastic Period, Mostagedda
High quality enlarged images of funerary wrapping fragments supported the supposition that resin was used in the preparation of the body in many Badarian and Predynastic interments. The specimen shows bone (white), skin, desiccated body tissue and linen (dark brown). Epi-illumination.

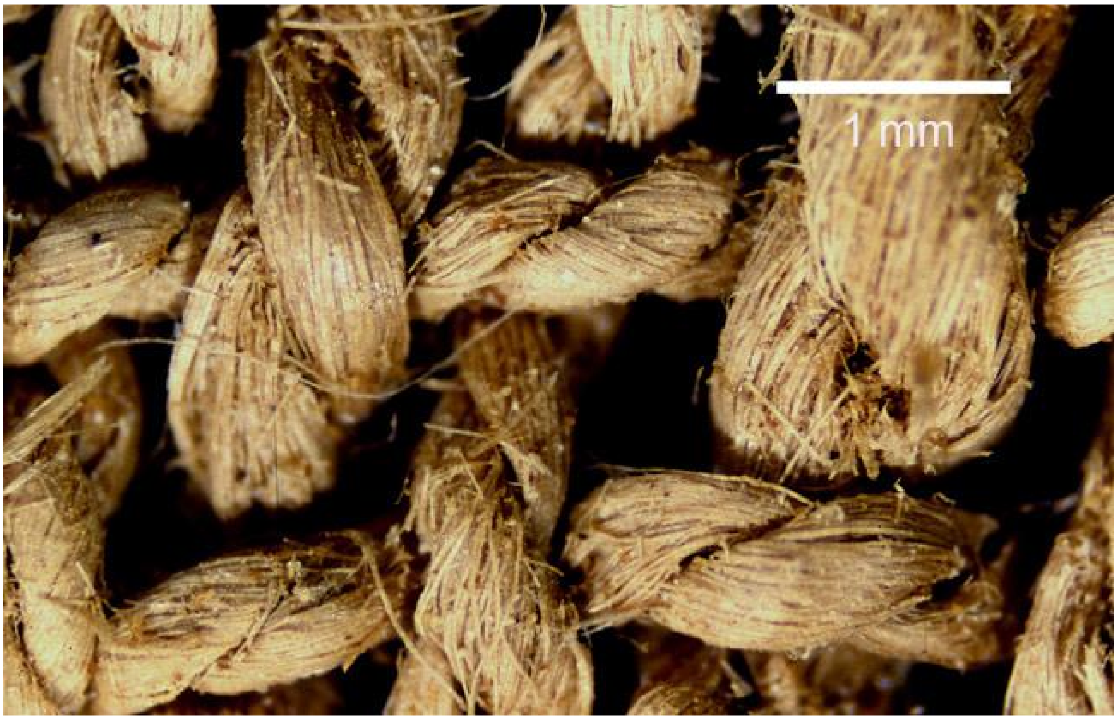

Figure S2: 33.30.85 Tomb No. 1223, Badarian Period, Mostagedda
Some textile fragments were quite clean, suggesting that they may have been used in the outer wrappings that were not in direct contact with the body. Epi-darkfield (HD) illumination.

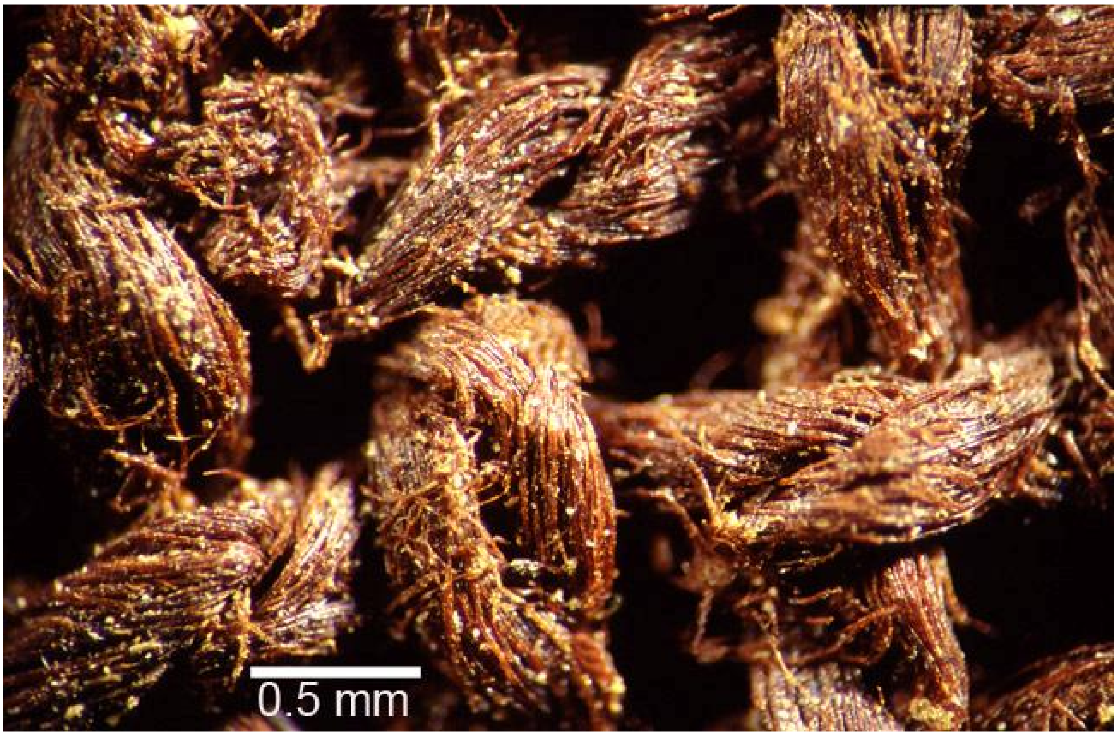

Figure S3: 33.30.91 Tomb No. 1206 B, Badarian Period, Mostagedda
Other textiles show the yarns to have a honey-brown ‘glaze’, presumed to be ‘resin’.
Epi-darkfield (HD) illumination.

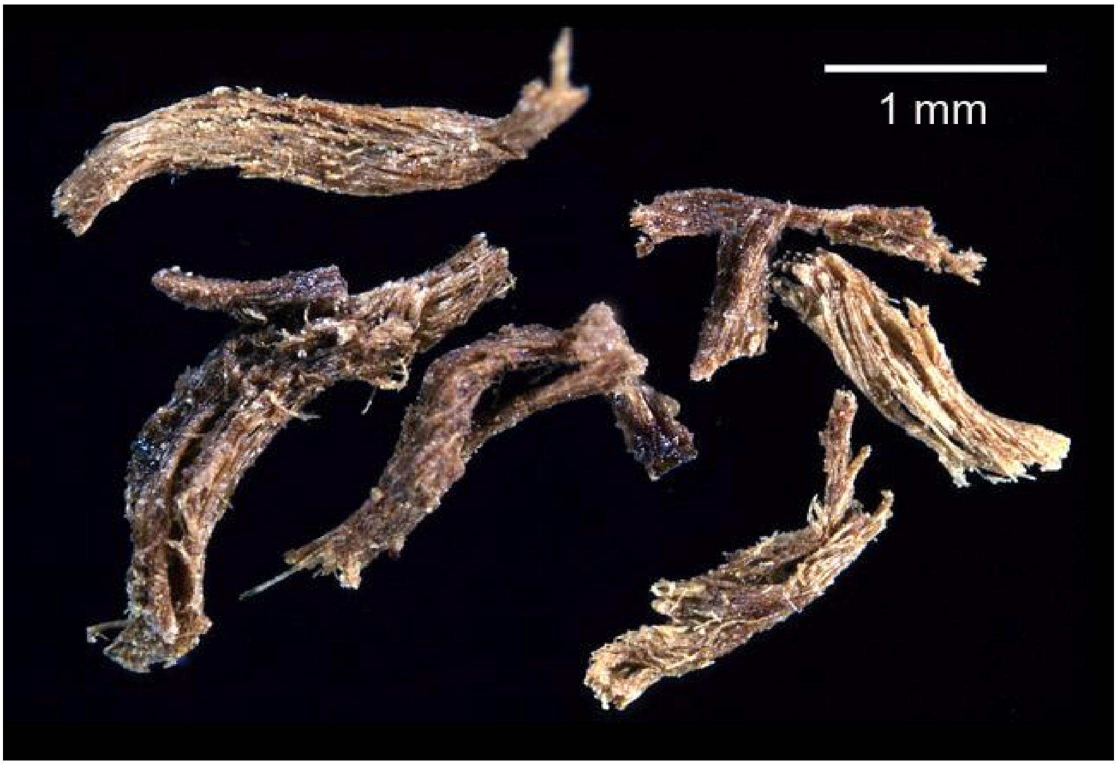

Figure S4: 33.30.44 Tomb No. 3538, Badarian Period, Mostagedda
Some textile remnants were very fragmentary, but even these sometimes showed the possibility of the presence of ‘resin’. Samples that presented themselves as likely to have ‘resin’ were examined macroscopically and then in the microscope. Epi-darkfield (HD) illumination.

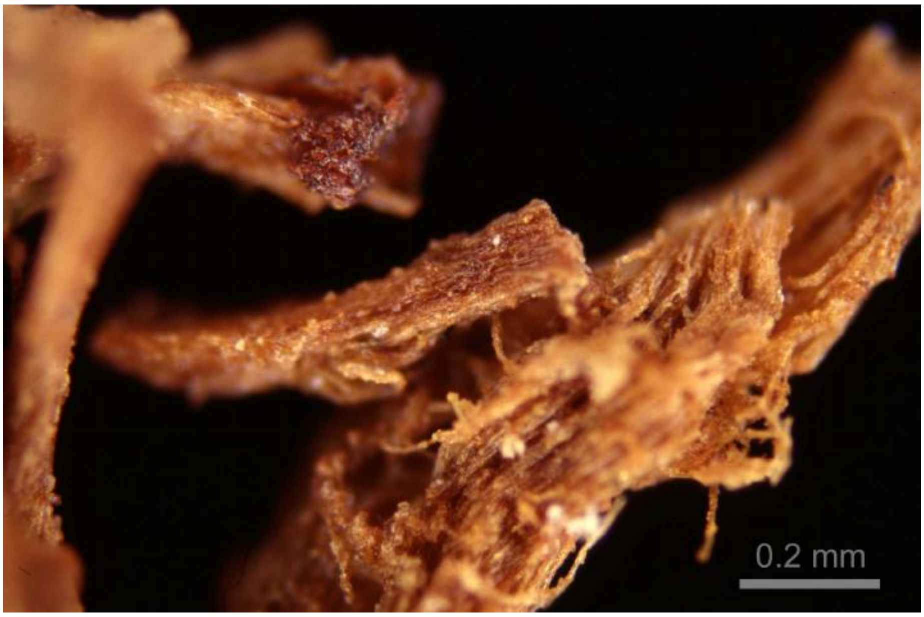

Figure S5: 33.30.44 Tomb No. 3538, Badarian Period, Mostagedda
The most convincing depiction of the 'toffee-like' (presumed) 'resin' was effected in the light microscope using HD illumination.

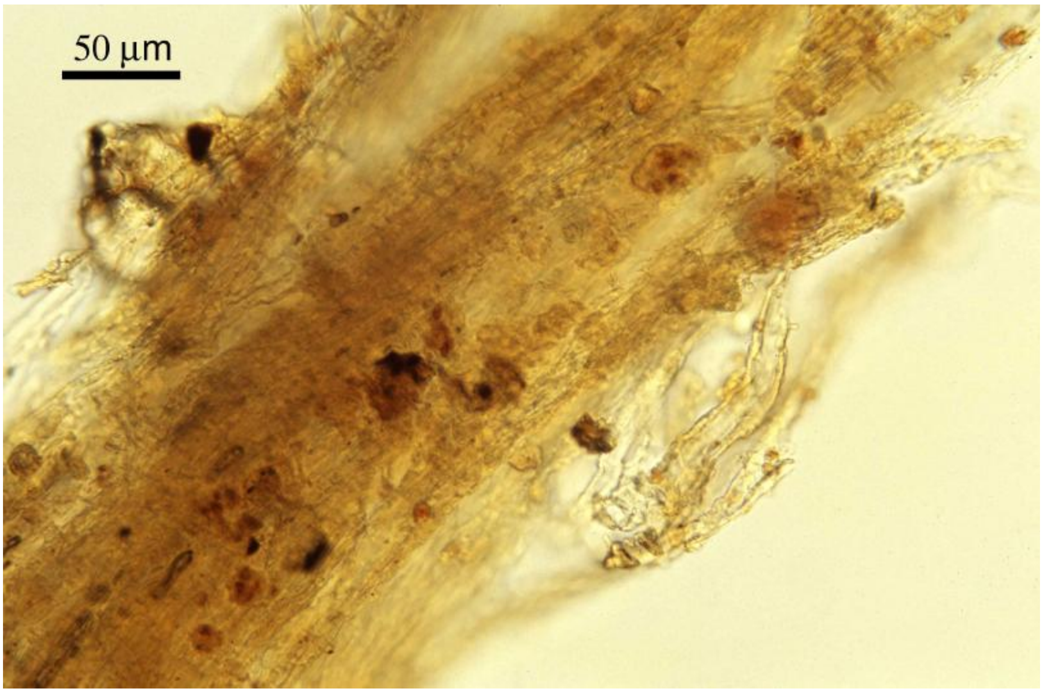

Figure S6: 33.30.44 Tomb No. 3538, Badarian Period, Mostagedda
Brightfield images were first routinely examined to look for promising areas for observation. Of the various microscopical techniques, crossed polars was the most revealing (Fig. 7). The manual interchange brightfield/crossed polars is quick and simple.

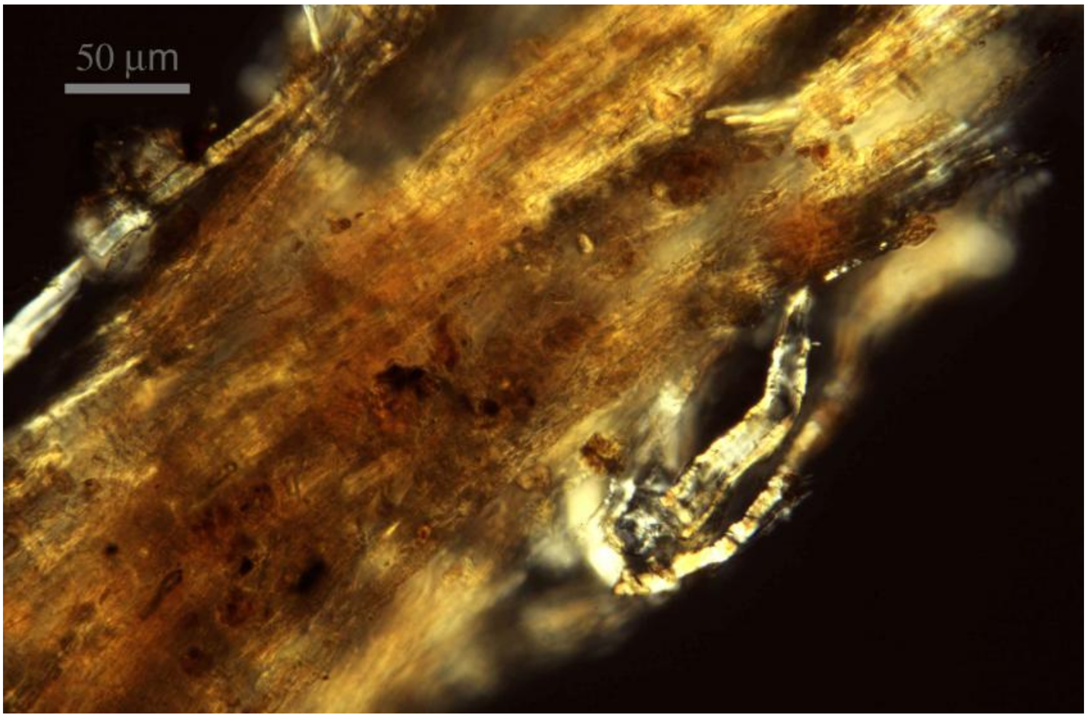

Figure S7: 33.30.44 Tomb No. 3538, Badarian Period, Mostagedda
The same field as Fig. 6, but with crossed polars. The polarisation colours are masked here by the golden hue of the resinous substance coating the fibers. Some typical flax fibers are visible on either side of the mass of ultimates.

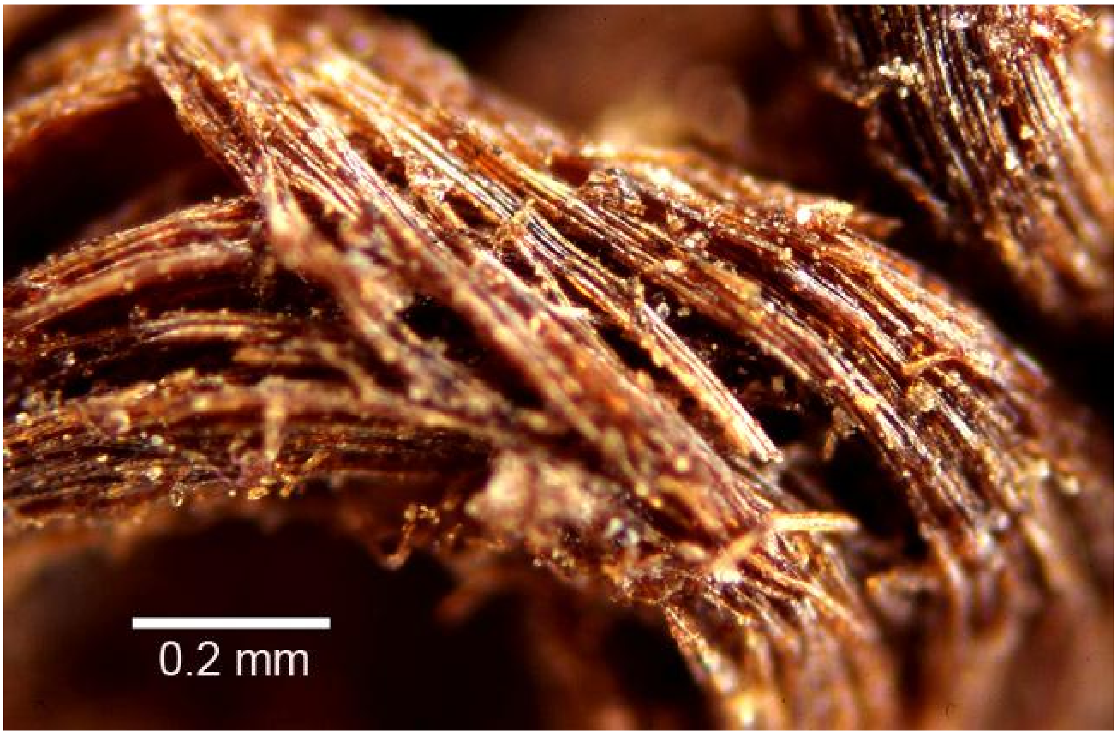

Figure S8: 33.30.80 Tomb No. 1215, Badarian Period, Mostagedda
Epi-darkfield (HD) illumination permits extremely high contrast and excellent relief to reveal the individual ‘resin’ impregnated fibers.

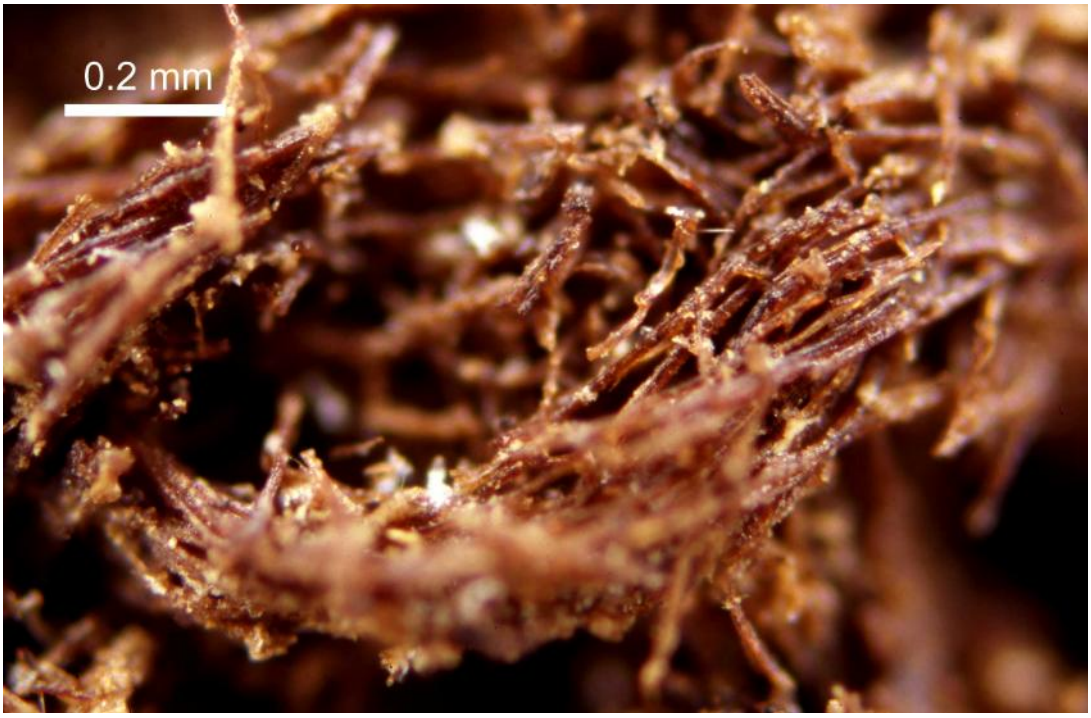

Figure S9: 33.30.83 Tomb No. 1214, Badarian Period, Mostagedda
Epi-darkfield (HD) illumination. A sector stop in the light path together with a rotating stage has been used to accentuate the relief. The limited depth of field is a major disadvantage of the technique.

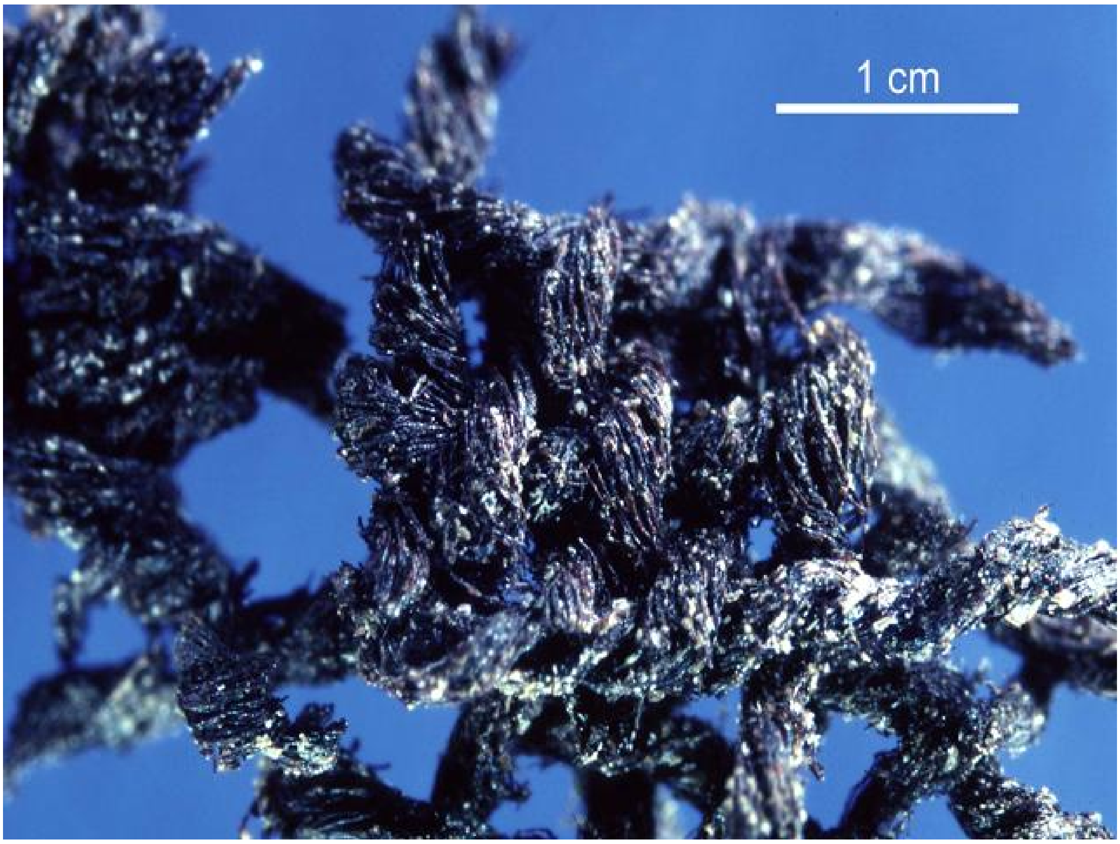

Figure S10: 33.30.92 Tomb No. 1637, Predynastic Period, Mostagedda
Although it appeared to be heavily carbonized this textile fragment, when enlarged, gave visual evidence of presumed ‘resin’. The s-spin typical of Predynastic yarns is shown to advantage. Epi-darkfield (HD) illumination.

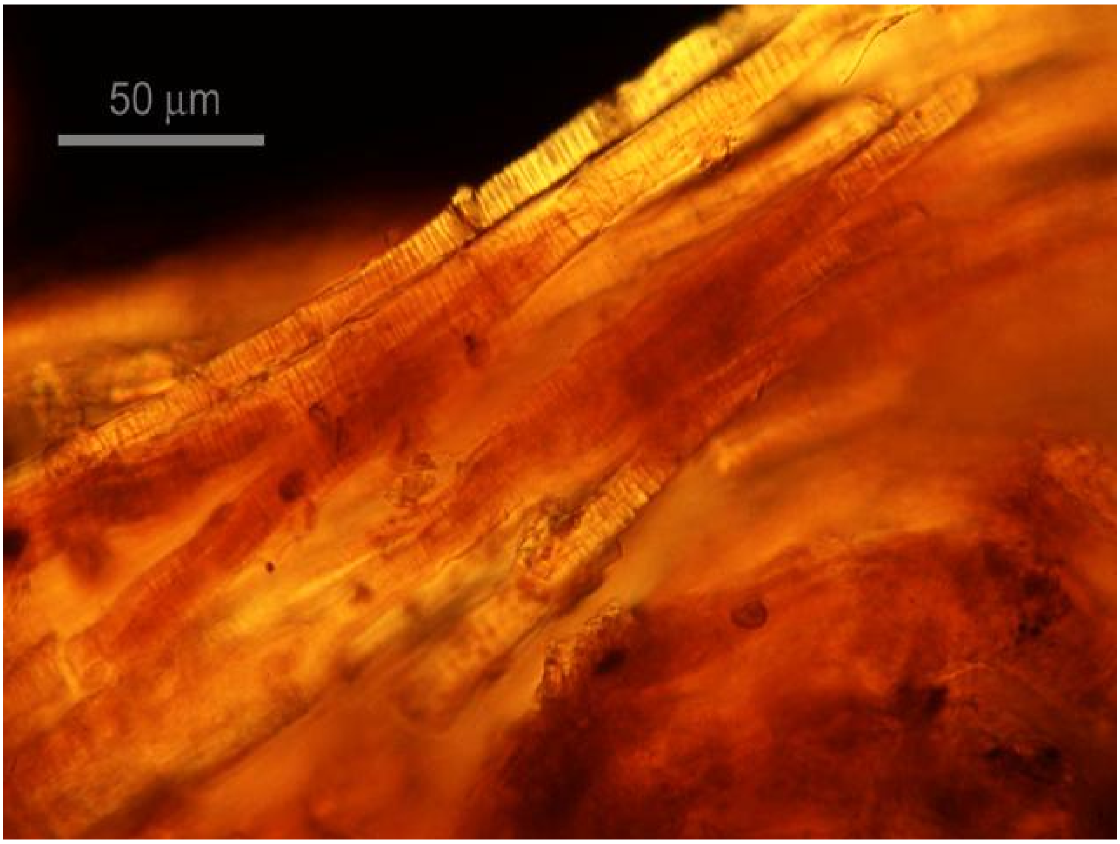

Figure S11: 33.30.92 Tomb No. 1637, Predynastic Period, Mostagedda
The separation of phloem fiber (‘flax’) from the surrounding stem tissue was generally very efficient, but in this resinous mass, fiber-tracheids from the adjacent xylem are abundant. Crossed polars.

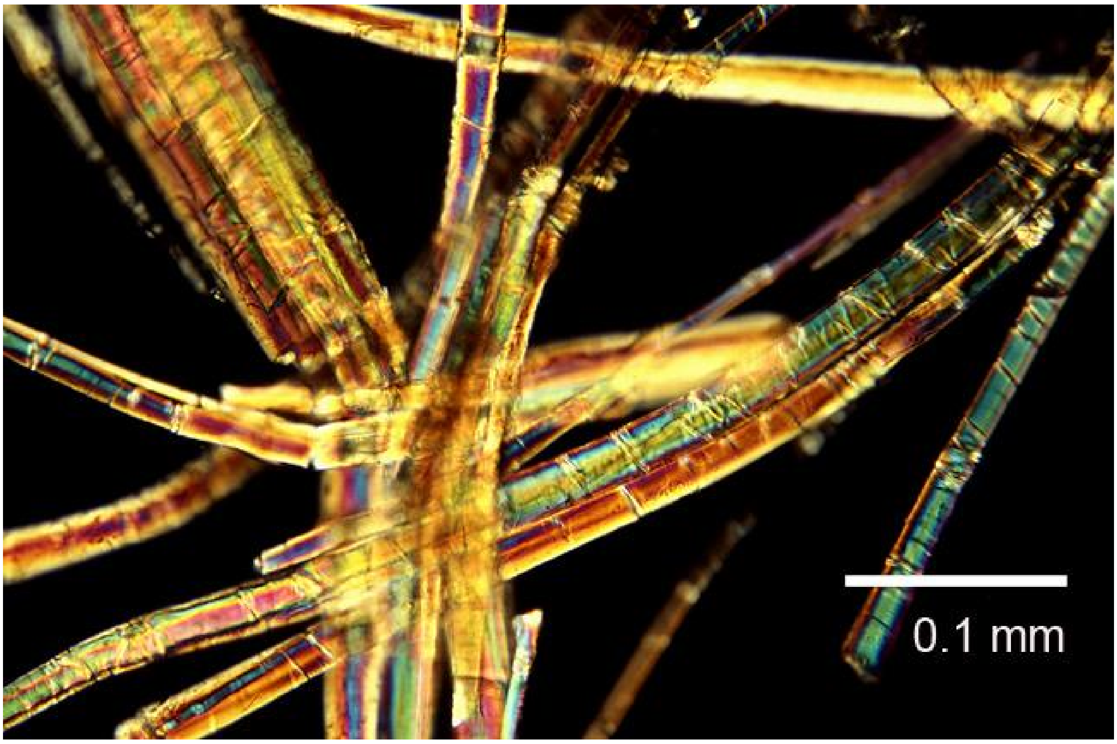

Figure S12: 56.30.1 Tomb 1800, Early Predynastic Period, Mostagedda
This image shows the typical features of flax: frequent cross-markings, small central lumen and low polarisation colours. The colours are characteristic of the thickness of individual fibers, orientation with respect to the polars, and birefringence (an optical property) of the cellulose. Crossed polars.

AMS radiocarbon dating

Samples of textile were dated at the Oxford Radiocarbon Accelerator Unit (ORAU), University of Oxford. The samples were treated with a dilute acid wash (0.5M HCl at RT for 30 mins), rinsed and dried. Combustion of the treated samples was achieved using a Europa Scientific ANCA-MS system, comprising a 20-20 IR mass spectrometer linked with a Roboprep or Carlo-Erba CHN sample converter unit. This operates in a continuous flow mode with a He carrier gas. $\delta^{13}C$ values are reported with reference to VPDB. Graphitisation of CO_2 was achieved using the standard ORAU method that is outlined in Dee and Bronk Ramsey [4]. The Oxford AMS radiocarbon instrumentation is described by Bronk Ramsey *et al.* [5]. Radiocarbon dates are expressed as conventional radiocarbon ages BP after Stuiver and Polach [6].

OxA-X	Sample	Material	CRA	+/-	fM	+/-	$\delta^{13}C$ (‰)
2212-36	33.30.44.1 Not Ex	Textile	4084	31	0.60144	0.00231	-24.9
2213-52	33.30.80.2 Not Ex	Textile	3787	30	0.62414	0.00234	-24.9
2213-53	33.30.83.2 Not Ex	Textile	4779	32	0.55161	0.00218	-23.7
2213-55	33.30.92.1 Not Ex	Textile	3867	31	0.61795	0.00237	-24.2

Table S2: Conventional radiocarbon ages (CRAs) determined for the 4 textile samples from Mostagedda. Samples are given OxA-X results to denote their non-routine chemistry at ORAU. fM is fraction modern. $\delta^{13}C$ values are given in ‰ with respect to VPDB.

The radiocarbon samples we dated were from textile samples that were later shown to have proportions of both modern and ^{14}C-free exogenous carbon in them derived from conservation attempts on the textiles. We used GC/MS methods to determine the proportions of these additives. They included Zellon, consisting of 95% cellulose acetate and 5% additives (plasticisers, specifically (phthalates), and chlorinated aromatics to allow greater penetration of the fibers). We know that Zellon was used on the Mostagedda textiles. The additives identified are typical of cellulose acetate formulations. Two of the samples contained products derived from natural petroleum seep. As such these would be expected to contain no ^{14}C.

We partitioned the carbon sources based on the GC/MS results to being either modern (which we estimate to be equivalent to 1 fM (fM is fraction modern, with 1 fM = 1950 AD) or ancient/^{14}C free (which we take to mean a value of 0 fM (background or >than ~55-60 000 BP). The modern contaminants were found to consist of plasticisers and cellulose acetates in the Zellon. The radiocarbon dead contaminants were derived from plasticizers, petroleum seep products and chlorinated aromatics. We recalculated the radiocarbon activities (expressed in fraction modern, fM) by measuring the proportion of these old and new contaminants in the treated textile samples and thereby corrected our AMS dates to account for these exogenous compounds.

If c = the proportion of modern (fM=1) C contamination in the textile, b = the proportion of original carbon in the textile, A_m = the measured textile radiocarbon age in fM and a = the proportion of old (fM=0) C contamination, we can calculate the correct radiocarbon age as:

$$(a \times fM0) + (b \times A_m) + (c \times fM1) \qquad (1)$$
$$= (a + b + c)A_m \qquad (2)$$
$$=(A_m - c)/b \qquad (3)$$

Using equation (3), we can calculate the new fM, which we term fM_{corr}.

We calculate a corrected radiocarbon age BP using -8033*ln(fM_{corr}).

We use the following equation to increase the error terms on the determinations:

$$= fM_{corr} \times \sqrt{}((fM_\sigma/(A_m\text{-}c) \times (fM_\sigma/(A_m\text{-}c)) + (c_\sigma/(A_m\text{-}c)) \times (c_\sigma/(A_m\text{-}c)) + (a_\sigma/A_m) \times (a_\sigma/A_m)$$

Where c_σ is the estimated uncertainty on the calculated proportion of the modern contaminant (est. 5%) and b_σ is the estimated uncertainty on the total C mass (assuming an error of ±3.6% for the error on the ancient carbon mass and ±5% for the error on the modern contamination). fM_σ is the standard error on the original AMS measurement (Table S3 col. 5). The measurement error terms in Table S2 are increased in the light of these recalculations.

OxA-X	Corrected age BP	±	fM	Corrected fM error	Calibrated age BC (68.2% range)		Calibrated age BC (95.4% range)	
					from	to	from	to
2212-36	5300	57	0.516960388	0.003657001	4231	4047	4316	3986
2213-52	4717	49	0.555893036	0.003357772	3630	3378	3635	3372
2213-53	5123	36	0.528474395	0.002384288	3974	3812	3989	3800
2213-55	4450	41	0.574662705	0.002925416	3325	3024	3339	2933

Table S3: Corrected radiocarbon ages BP and errors. Ages are calibrated using the INTCAL13 curve [7]. The original ages are shown in Table S2 Calibrated ages are rounded to the nearest 10. See Figure S13 for an illustration of the calibrated data.

The first three determinations are on textiles dated to the Badarian period (currently estimated by Dee et al. to start between ~4489-4266 BC at 95.4% probability and end between 3896-3616 BC) (Figure S13). Two of the re-corrected results are entirely

consistent with this estimate (OxA-V-2212-36 and OxA-V-2213-53). Another is marginally young (OxA-V-2213-52) and here we may have underestimated the amount of contamination in the textiles. The final sample is of a textile dating to the Naqada IIC period. For the subperiod Naqada IIC-IID2 we would expect the age to start between about 3562-3367 BC (transition IIB to IIC) and end prior to 3377-3238 BC (the transition from IID to IIIA) In Fig S13 we use a range from 3450-3300 BC for the phase. The recalculated result is therefore in good agreement with the archaeological age estimate.

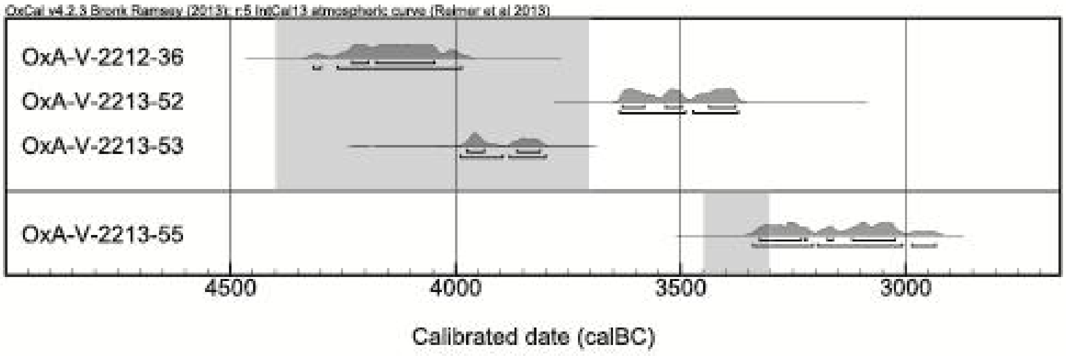

Figure S13: Calibrated ages for the corrected textile dated samples. See Table S3 for the data corresponding to these probability distributions at 68.2% and 95.4% probability. The shaded areas denote the accepted archeological age range for the Badarian (top of picture) and Naqada IIC period (bottom) respectively. (Figure generated using OxCal 4.1[5]).

Chemical analyses

As discussed in the main text, the amorphous organic residues associated with Badarian and Predynastic funerary wrappings and hypothesized to be resin were chemically characterized and identified using a dual analytical approach of conventional gas chromatography (GC-MS), following solvent extraction procedures, and sequential thermal desorption-gas chromatography-mass spectrometry (TD-GC-MS) and pyrolysis-gas chromatography-mass spectrometry (Py-GC-MS). This allowed the chemical analysis of both free and bound/polymerized biomarkers likely to be present [11].

Experimental

Sample preparation for Gas chromatography-mass spectrometry (GC-MS)

The samples were initially ground to a fine powder. A weighed amount of these ground samples (from ~0.5-25mg depending on sample available) was taken, and where appropriate an internal standard was added for quantification (10-100 µg of tetratriacontane, n-C_{34} alkane). These samples were then extracted with an appropriate volume (0.1-1mL) of chloroform-methanol solution (2:1 v/v; 3 x 60 min sonication). After centrifugation (20 min, 1000 rpm) the supernatant solvent was removed from the residue and placed in a vial. The three extracts were combined and the solvent reduced by rotary evaporation. Following transfer of the combined extracts to a screw-capped vial, the remaining solvent was removed by evaporation under a gentle stream of nitrogen at 40°C. The residue was reweighed to give total lipid extracts (TLE). Free hydroxyl and carboxylic acid groups from the TLE were derivatized to their corresponding trimethylsilyl (TMS) ethers and esters, respectively, using *N,O*-bis(trimethylsilyl)trifluoroacetamide (Sigma-Aldrich Chemical Co., St Louis, MO, USA) containing 1% of trimethylchlorosilane (30-50 µl, 70°C, 1 hour). Excess BSTFA was then removed under a gentle stream of nitrogen and the derivatized sample redissolved in dichloromethane and analyzed by GC and GC-MS.

Gas chromatography (GC)

GC analysis of the total lipid extract of each sample was performed on a Hewlett-Packard 7890 gas chromatograph fitted with a split injector (325°C). The flame ionisation detector (FID) was set at a temperature of 350°C. Separation was performed on a fused silica capillary column (30m x 0.25mm i.d.) coated with 0.25µm 5% phenyl methyl polysiloxane (DB-5). Initially the GC oven was held at 40°C for 5 min and then temperature programmed from 40°C-350°C at 8°C/min and held at final temperature for 20 min, total 64 min. Hydrogen was used as the carrier gas (1mL/min, initial pressure of 45kPa, splitless injection 1 min).

Gas chromatography-mass spectrometry (GC-MS)

GC-MS analysis of the total lipid extract of each sample was performed on 1. a Hewlett-Packard 7890 gas chromatograph fitted with a split injector (325°C) interfaced to a Waters GCT Premier mass spectrometer (electron voltage 70eV, filament current 200µA, source temperature 170°C, interface temperature 325°C). The MS was set to scan in the range 40-850 amu. Separation was performed on a fused silica capillary column (30m x 0.25mm i.d.) coated with 0.25um 5% phenyl methyl polysiloxane (DB-5). Initially the GC was held at 40°c for 5 min and then temperature programmed from 40°C-350°C at 8°C/min and held at final temperature for 20 min, total 64 min, with Helium as the carrier gas (constant flow 1ml/min, initial pressure of 45kPa, splitless injection 1 min), and 2. a Hewlett-Packard 5890 Series II gas chromatograph fitted with a split injector (325°C) interfaced to a Trio 1000 mass spectrometer (electron voltage 70eV, filament current 200uA, source temperature 170°C, interface temperature 325°C). The MS was set to scan in the range 40-850 amu. Separation was performed on a fused silica capillary column (30m x 0.25mm i.d.) coated with 0.25um 5% phenyl methyl silicone (DB-5). Initially the GC was held at 40°C for 5 min and then temperature programmed from 40°C-350°C at 8°C/min and held at final temperature for 20 min, total 64 min, with Helium as the carrier gas (flow 1mL/min, initial pressure of 45kPa, splitless injection 1 min.) Peaks were identified on the basis of both their mass spectra (NIST Mass Spectral Database and additional data referenced below [12-27]), and retention times.

Sequential thermal desorption-gas chromatography-mass spectrometry (TD-GC-MS) and pyrolysis- gas chromatography-mass spectrometry (Py-GC-MS)

TD/Py-GC-MS analysis of each 'resin' sample was performed on a CDS Pyroprobe 1000 (Chemical Data System, Oxford, PA, USA) unit fitted with a platinum coil probe for the thermal extraction of free biomarker compounds and the pyrolysis of bound/polymerized components from the ground samples (0.1-1mg of sample weighed using a microanalytical balance). The samples were loaded into quartz tubes plugged with solvent extracted glass wool. The quartz tubes were then inserted into the platinum coil of the probe which was inserted into the CDS1500 valved interface (320°C) connected to a Hewlett-Packard 5890 gas chromatograph split injector (280°C) interfaced to a Hewlett-Packard 5973MSD (electron voltage 70eV, filament current 220uA, source temperature 230°C, quadrupole temperature 150°C, multiplier voltage 2200V, interface temperature 340°C). The TD/Py temperature was held for 10 s. The samples were thermally desorbed at 310ºC, followed by pyrolysis at 610ºC. Separation was performed on a fused silica capillary column (30m x 0.25mm i.d) coated with 0.25µm 5% phenyl methyl polysilioxane (DB-5) stationary phase. Initially the GC oven was held at 35°C for 5 min and then temperature programmed from 35°C-340°C at 4°C/min and held at final temperature for 15 min, total 96 min, with Helium as the carrier gas (constant flow 1ml/min, initial pressure of 45kPa, split at 30 mL/min.). The acquisition was controlled by a HP kayak xa chemstation computer, in full scan mode (35-700 amu). Peaks were

identified on the basis of both their mass spectra (NIST Mass Spectral Database and additional data referenced below (12-27)) and retention times.

A total of 20 samples were analyzed in this way. Where there was little extractable material, and therefore not easily quantifiable, the GC-MS data has not been shown, although these samples were also entirely consistent with the other samples from the same burials in terms of both the natural products constituting the ancient recipes and the relative proportions of those natural products.

Given the number of constituents in the ancient recipes it is not possible to display all on the chromatograms (TICs), even if they are present in appreciable abundance and of significance for the research presented. Such significance is fully explained within the main text and figures, and the supplementary information (including references) presented here.

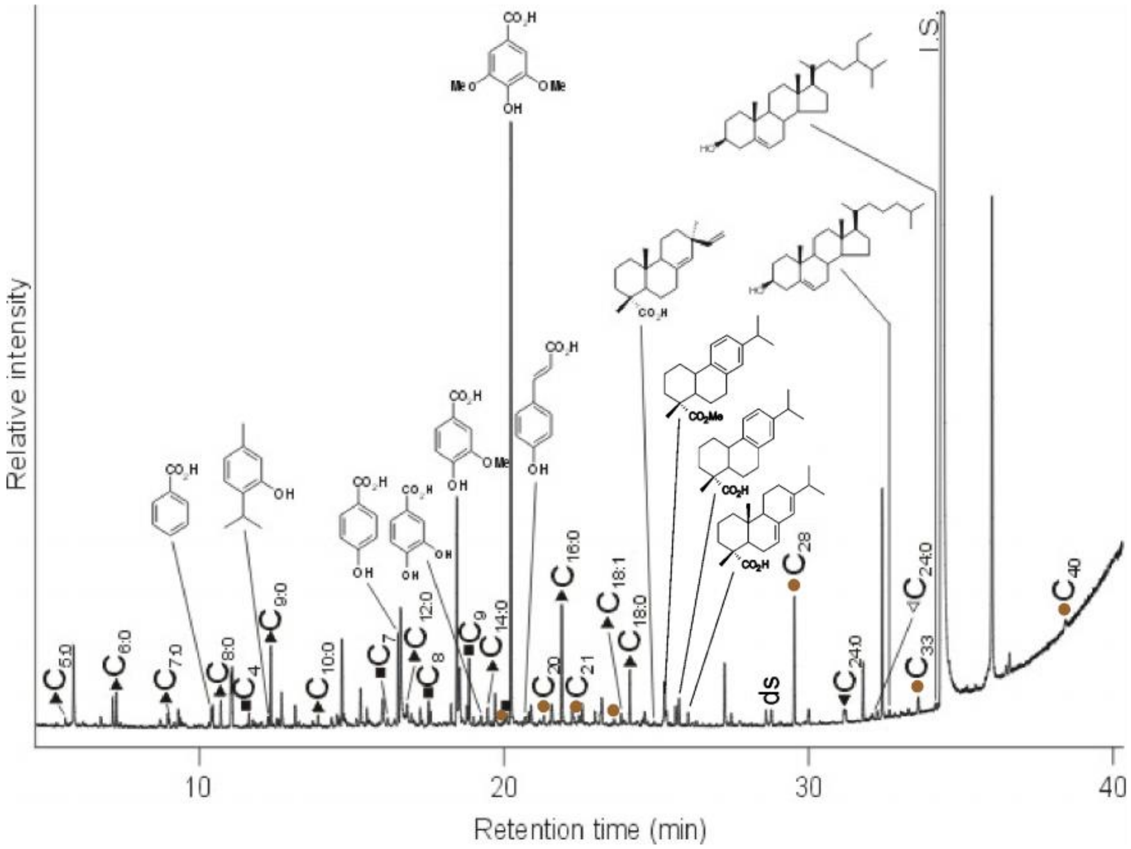

Figure S14 Reconstructed GC-MS TIC of the trimethylsilylated total lipid extracts of 33.30.44 3 (see Table 1). Peak identities as for Fig. 3 in main text. Additionally: brown circles, C_n indicates *n*-alkanes; also shown are the structures of two additional aromatic acids identified: pyrocatechuic acid (3,4-dihydroxybenzoic acid) and *p*-coumaric acid (4-hydroxycinnamic acid). IS, internal standard (n-tetratriacontane).

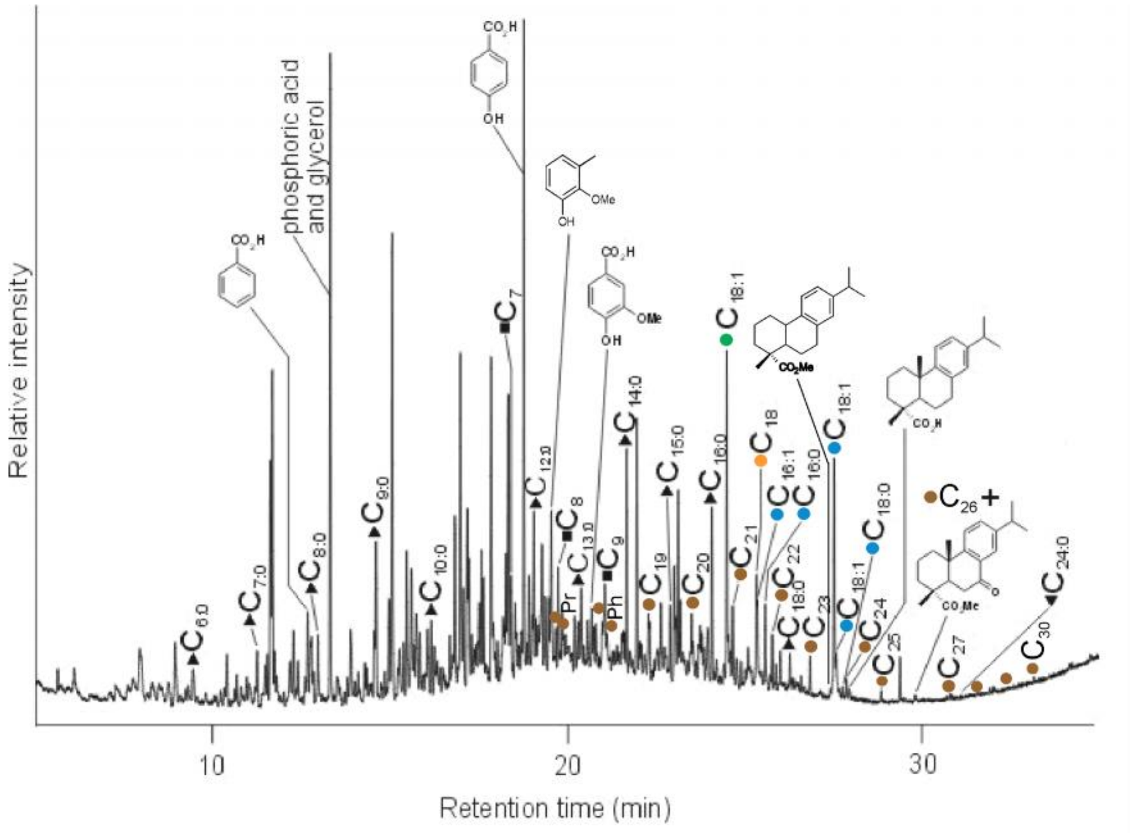

Figure S15 Reconstructed GC-MS TIC of the trimethylsilylated total lipid extract of 33.30.72 1 (see Table 2). Peak identities as for Fig. 3 in main text and Fig. S14. Additionally: green circles, $C_{n:i}$ indicates aliphatic nitrile; blue circles, $C_{n:i}$ indicates aliphatic amides; orange circle, C_n indicates *n*-alkanol; Pr and Ph next to the brown circles indicate the isoprenoid alkanes pristane and phytane respectively; also shown are phosphoric acid and glycerol and the structure of an aromatic phenol tentatively identified: 3-methyl guaiacol.

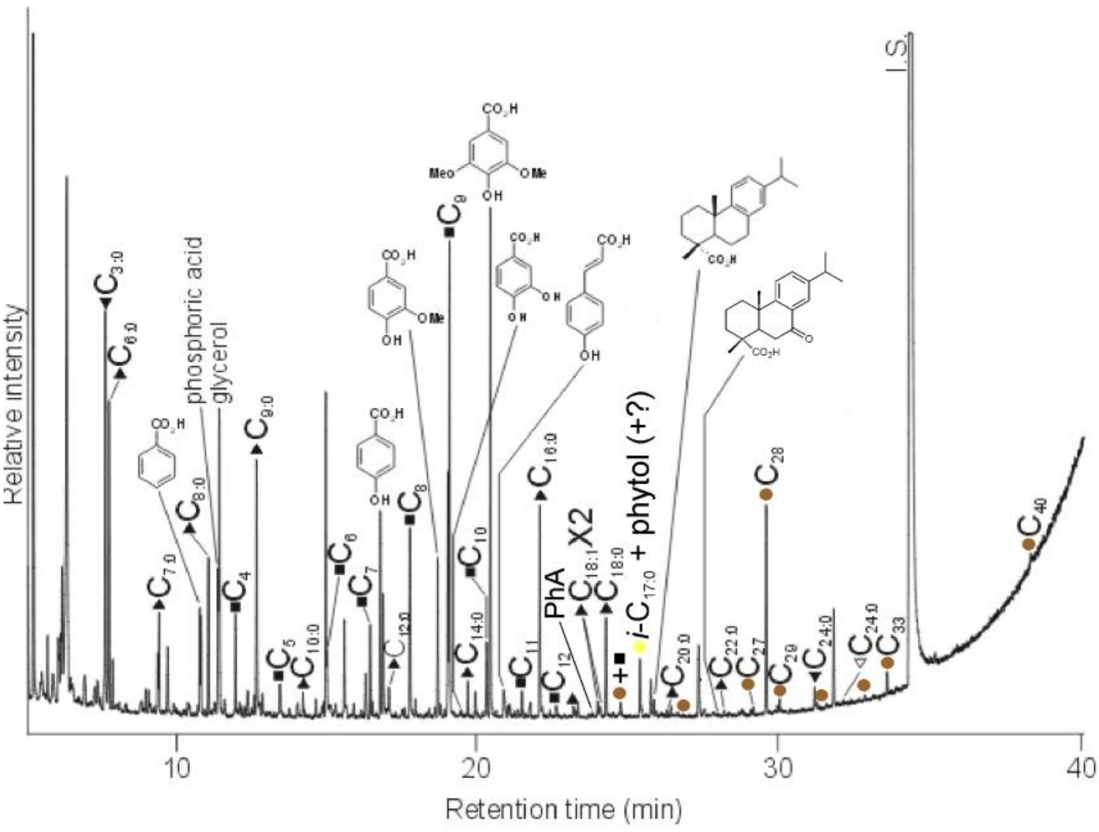

Figure S16 Reconstructed GC-MS TIC of the trimethylsilylated total lipid extract of 33.30.80 1 (see Table 2). Peak identities as for Figs. 3 in main text, S14 and S15. Additionally: PhA = phytanic acid; yellow circle, $C_{n:i}$ indicates 3-hydroxy fatty acid; which coeluted with phytol.

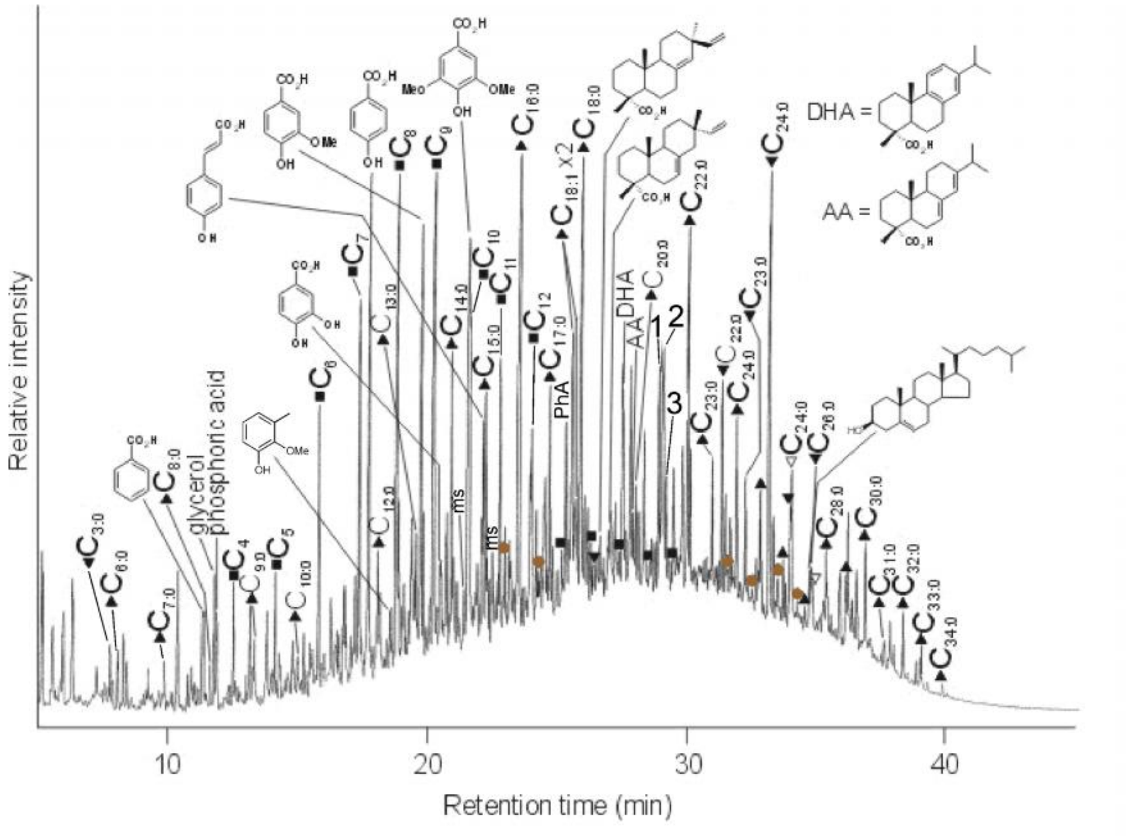

Figure S17 Reconstructed GC-MS TIC of the trimethylsilylated total lipid extract of 33.30.80 2 (see Table 2). Peak identities as for Fig.3 in main text and S14-S16. Additionally: ms = monosaccharides; the numbers 1-3 represent oxidized unsaturated fatty acids: 1 = 9,10-dihydroxyoctadecanoic acid (*threo* and *erythro* isomers), 2 = a 10,12-dihydroxyoctadecenoic acid, 3 = 10,12-dihydroxyoctadecanoic acid + 11,12-dihydroxyoctadecanoic acid.

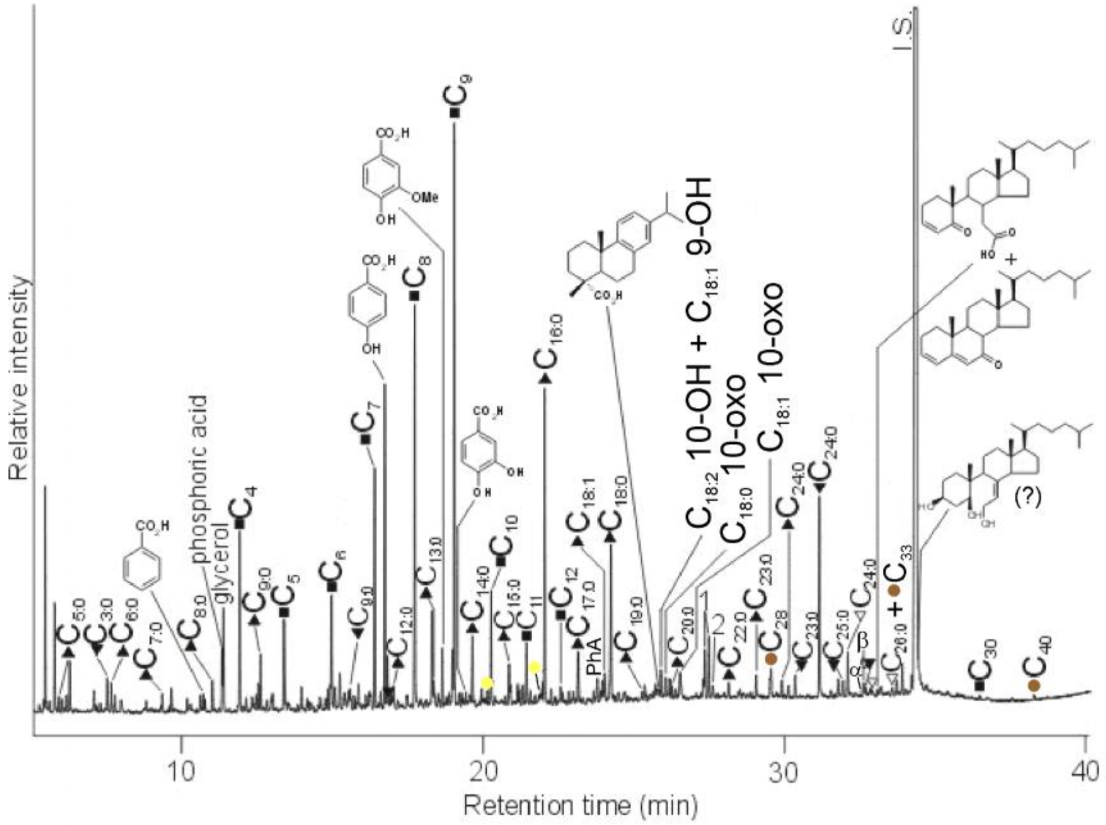

Figure S18 Reconstructed GC-MS TIC of the trimethylsilylated total lipid extract of 33.30.80 3 (see Table 2). Peak identities as for Fig. 3 in main text and S14-S17. The numbers 1-2 represent oxidized unsaturated acids: 1 = 9,10-dihydroxyoctadecanoic acid (*threo* and *erythro* isomers), 2 = 11,12-dihydroxyoctadecanoic acid; $C_{18:1}$ 10-oxo indicates a 10-oxo-octadecenoic acid and $C_{18:0}$ 10-oxo indicates 10-oxo-octadecanoic acid; $C_{18:1}$ 9-OH is a 9-hydroxy-octadecenoic acid and $C_{18:2}$ 10-OH is a 10-hydroxyoctadecadienoic acid. Also shown are three steroidal compounds identified: cholesta-3,5-dien-7-one, 5-oxo-5,6-secocholest-3-en-6-oic acid and hipposterol (5,6-secocholest-7-en-3β,5β,6-triol; tentatively identified). The letters α and β represent two further steroidal compounds identified: α = 5,6-secocholest-3,6/7-dien-5-one (very tentatively identified), β = cholesterol.

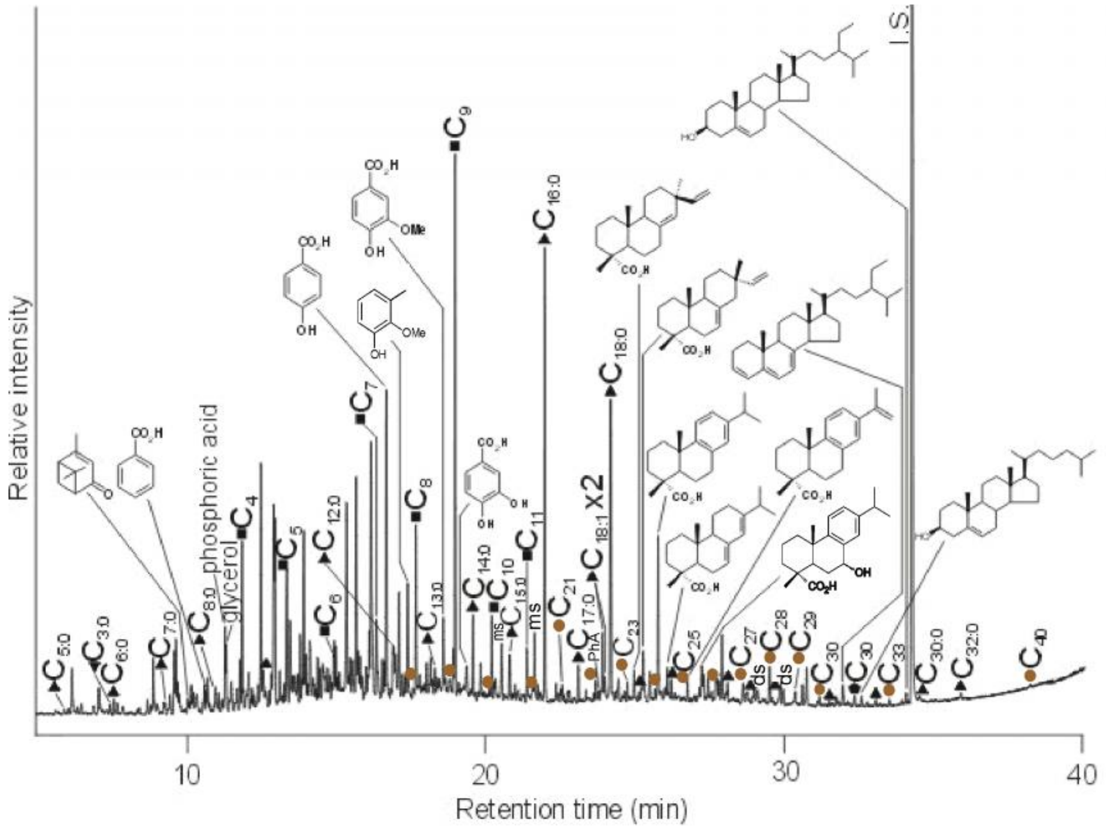

Figure S19 Reconstructed GC-MS TIC of the trimethylsilylated total lipid extract of 33.30.80 4 (see Table 2). Peak identities as for Fig 3 in main text and S14-S18. Also shown is the monoterpenoid identified: 2-pinen-4-one (verbenone), two additional diterpenoid acids identified are shown: 15-dehydrodehydroabietic acid and 7-hydroxydehydroabietic acid.

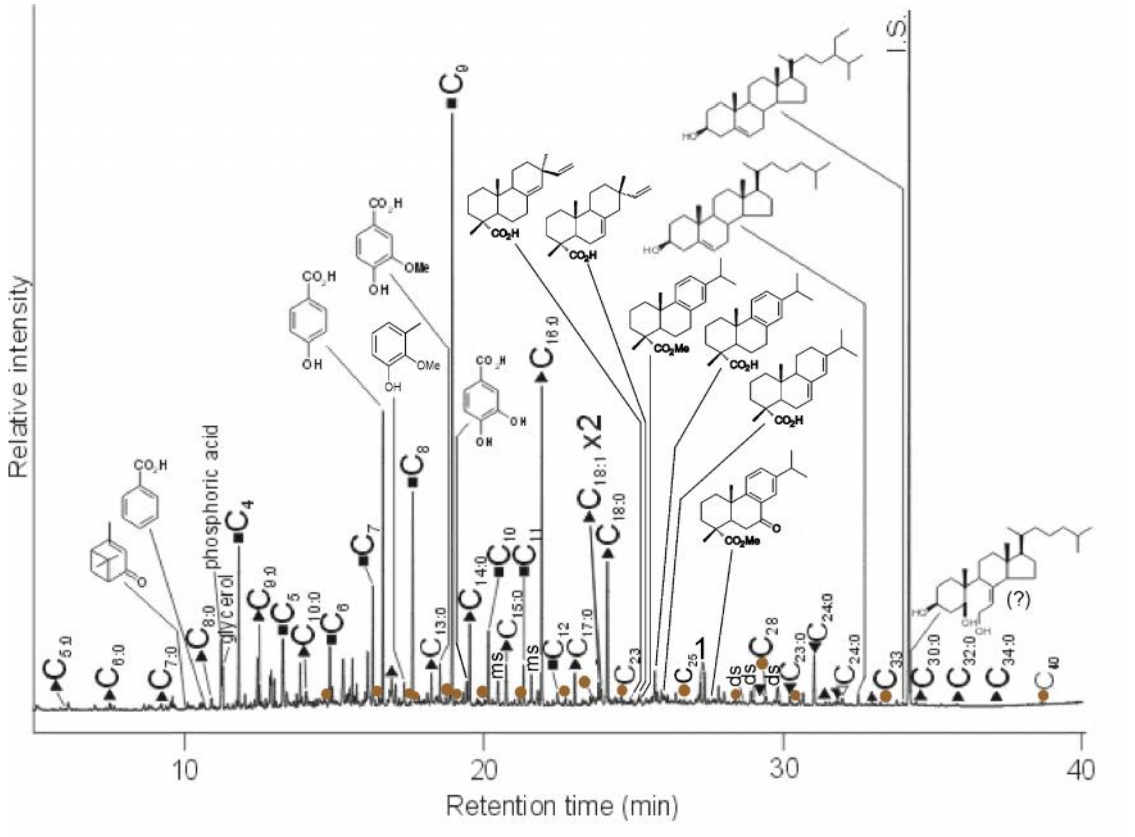

Figure S20 Reconstructed GC-MS TIC of the trimethylsilylated total lipid extract of 33.30.80 6 (see Table 2). Peak identities as for Fig. 3 in main text and S14-S19.

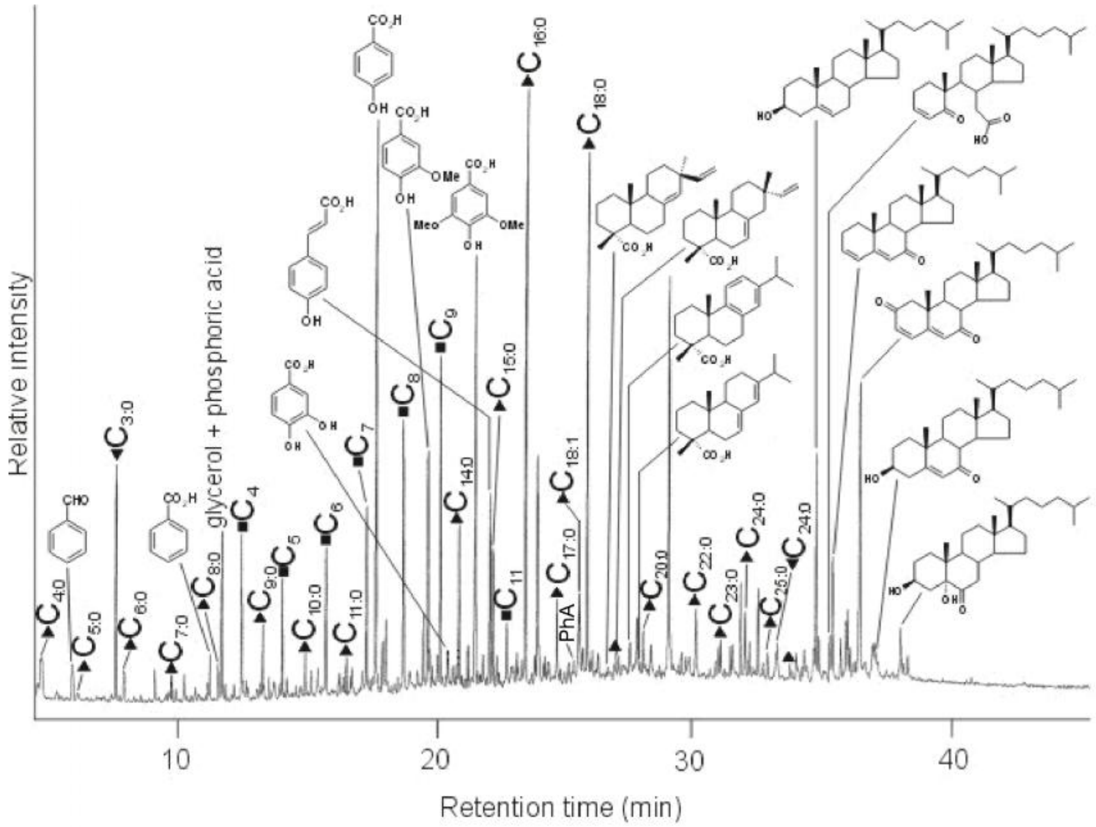

Figure S21 Reconstructed GC-MS TIC of the trimethylsilylated total lipid extract of 33.30.83 2 (see Table 2). Peak identities as for Fig. 3 in main text and S14-S20 below. Also shown is benzaldehyde and two additional steroidal compounds identified: cholesta-3,5-dien-2,7-dione and 6-oxocholestan-3β,5α-diol.

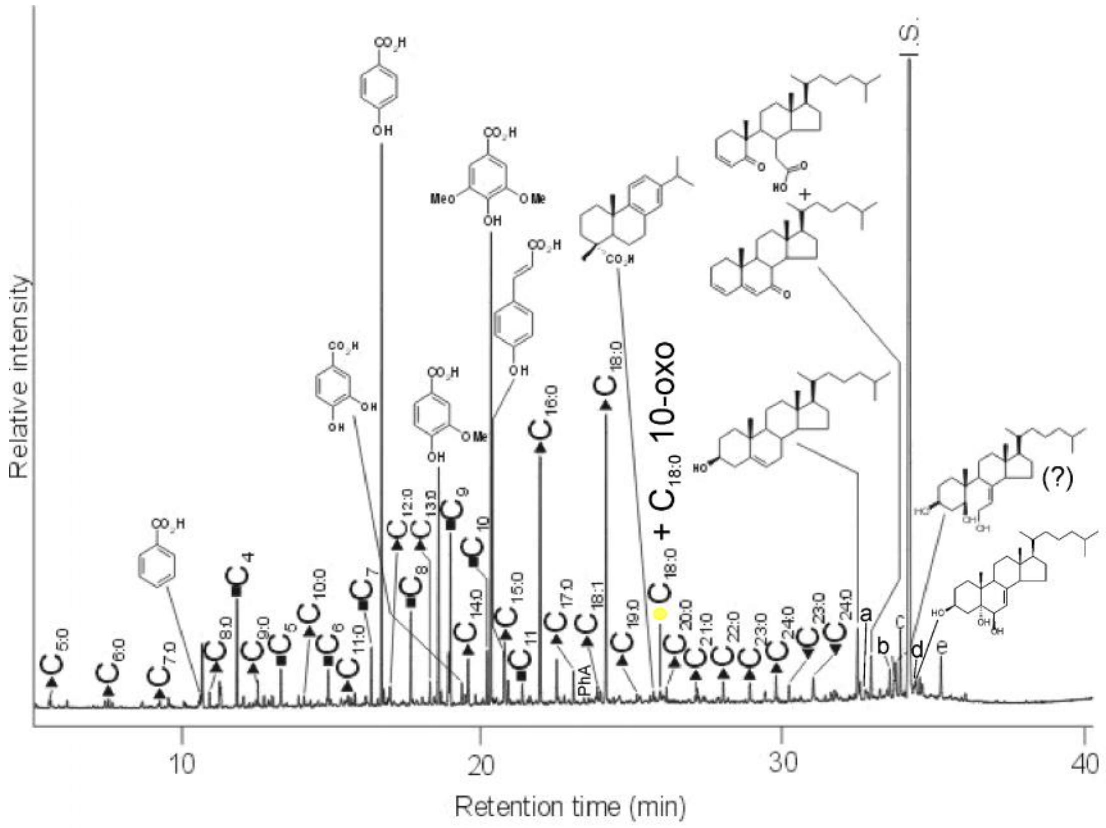

Figure S22 Reconstructed GC-MS TIC of the trimethylsilylated total lipid extract of 33.30.83 3 (see Table 2). Peak identities as for Fig. 3 in main text and S14-S21. Also shown is one steroidal compound identified: cholest-7-en-3β,5α,6β-triol. The letters a-e represent further steroidal compounds identified: a = cholest-4-en-3β,6α-diol, b = cholesterol-5α,6α-epoxide (cholesterol α-oxide), c = cholesta-3,5-dien-2,7-dione, d = cholestane-3β,5α,6α-triol, e = 6-oxocholestan-3β,5α-diol.

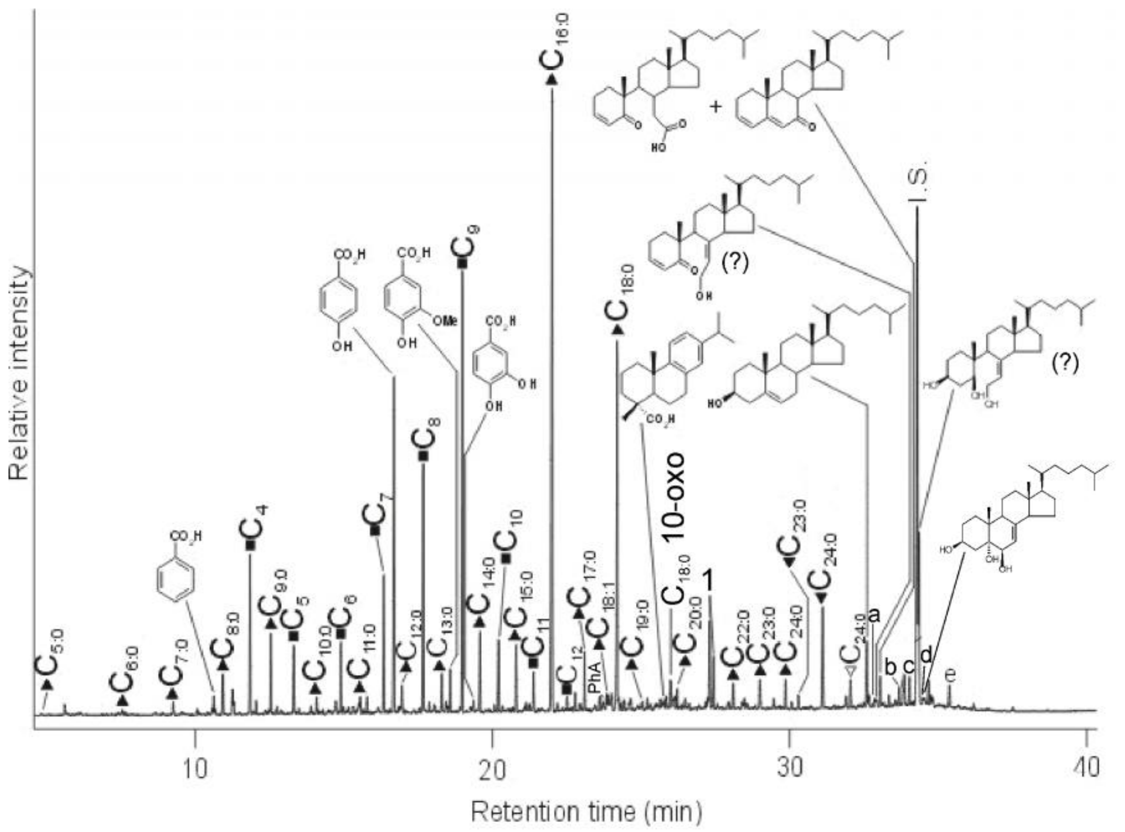

Figure S23 Reconstructed GC-MS TIC of the trimethylsilylated total lipid extract of 33.30.83 4 (see Table 2). Peak identities as for Figs. 3 in main text and S14-S22. Also shown is the steroidal compound: 5-oxo-5,6-secocholesta-3,7-diene-6-ol (very tentatively identified).

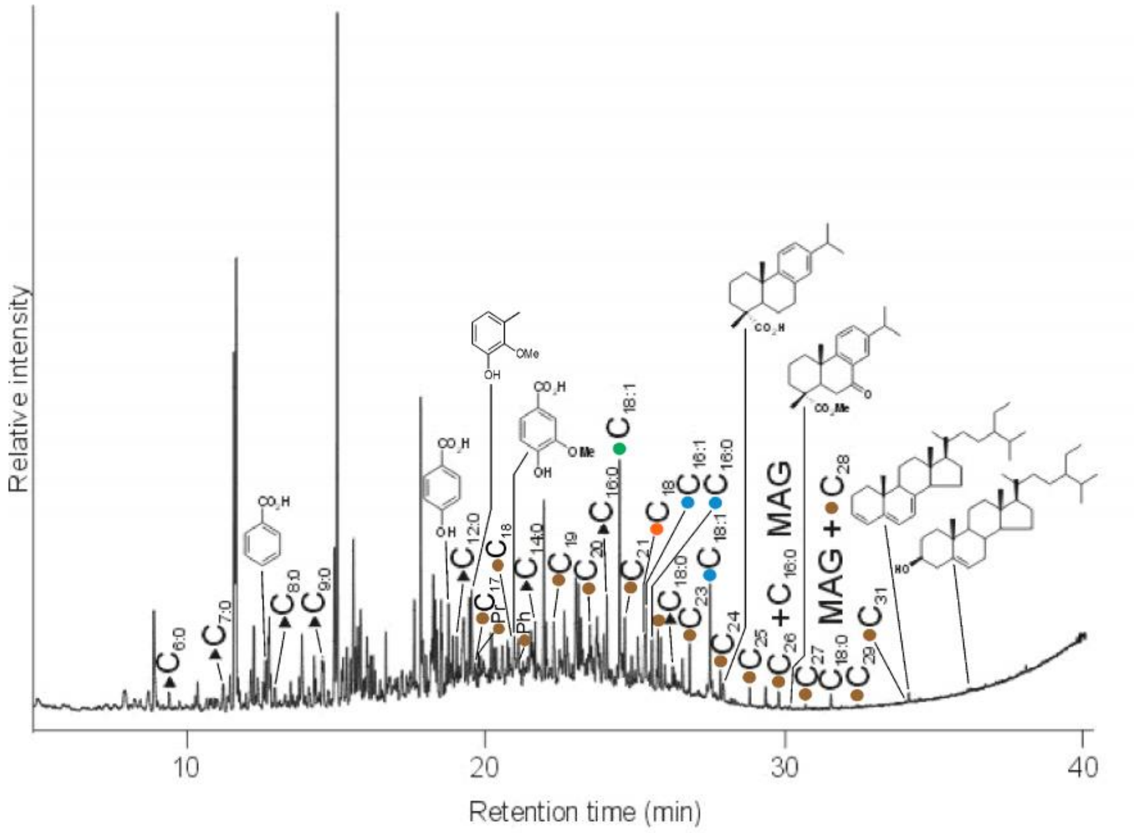

Figure S24 Reconstructed GC-MS TIC of the trimethylsilylated total lipid extract of 33.30.30 1 (see Table 2). Peak identities as for Fig. 3 in main text and S14-S23. Also shown are the palmityl ($C_{16:0}$) and stearyl ($C_{18:0}$) monoglycerides (labelled MAGs).

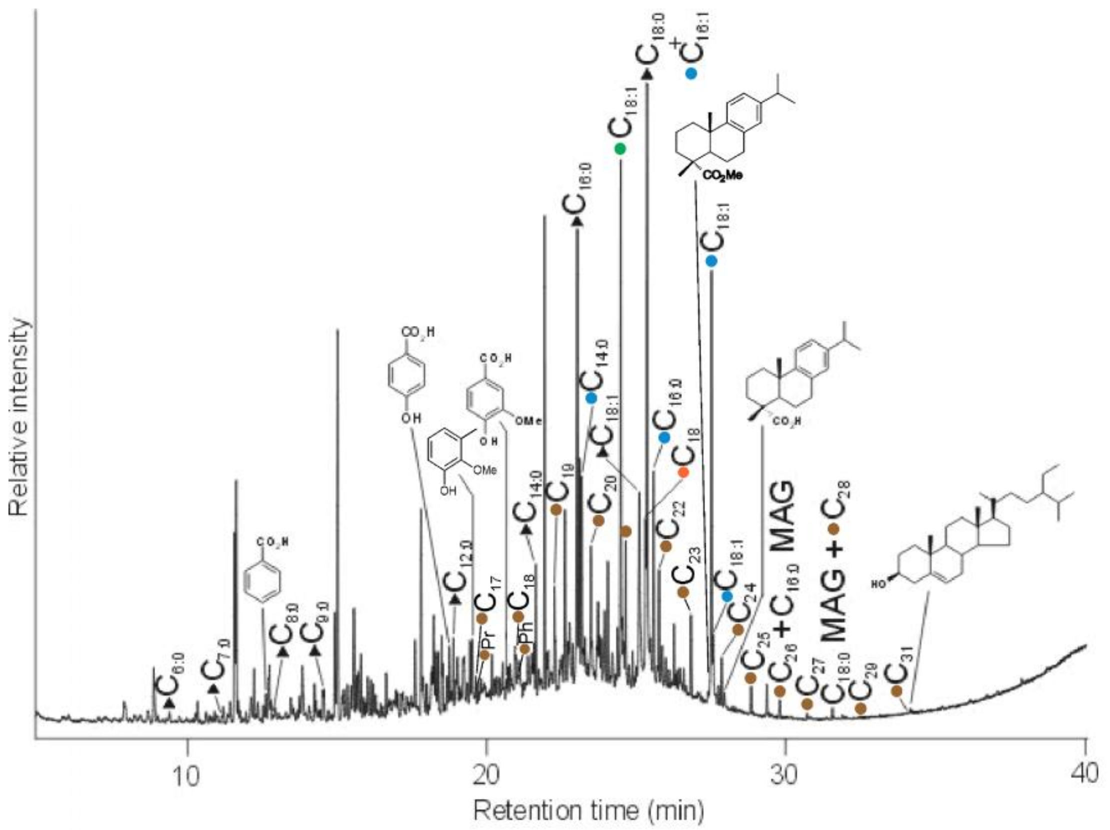

Figure S25 Reconstructed GC-MS TIC of the trimethylsilylated total lipid extract of 33.30.53 1 (see Table 2). Peak identities as for Fig. 3 in main text and S14-S24.

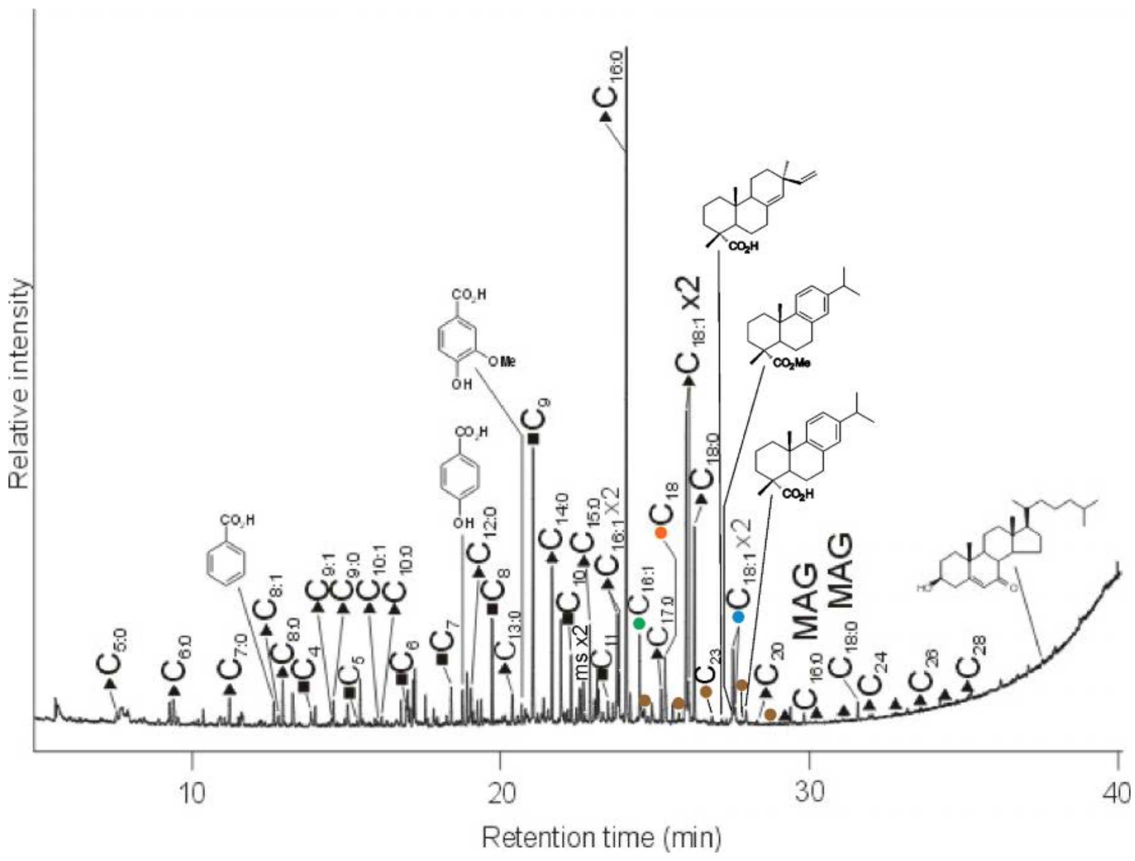

Figure S26 Reconstructed GC-MS TIC of the trimethylsilylated total lipid extract of 33.30.59 1 (see Table 2). Peak identities as for Fig. 3 in main text and S14-24.

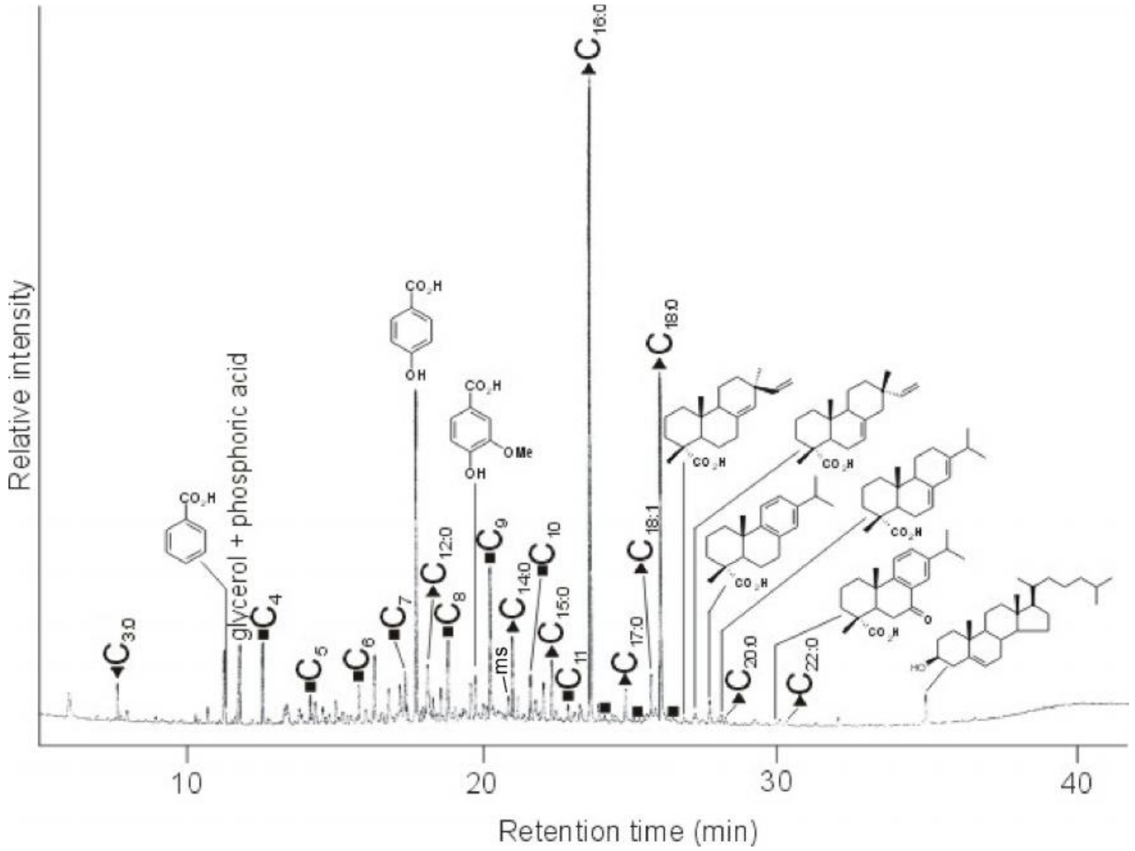

Figure S27 Reconstructed GC-MS TIC of the trimethylsilylated total lipid extract of 33.30.92 1 (see Table 2). Peak identities as for Fig. 3 in main text and S14-S26.

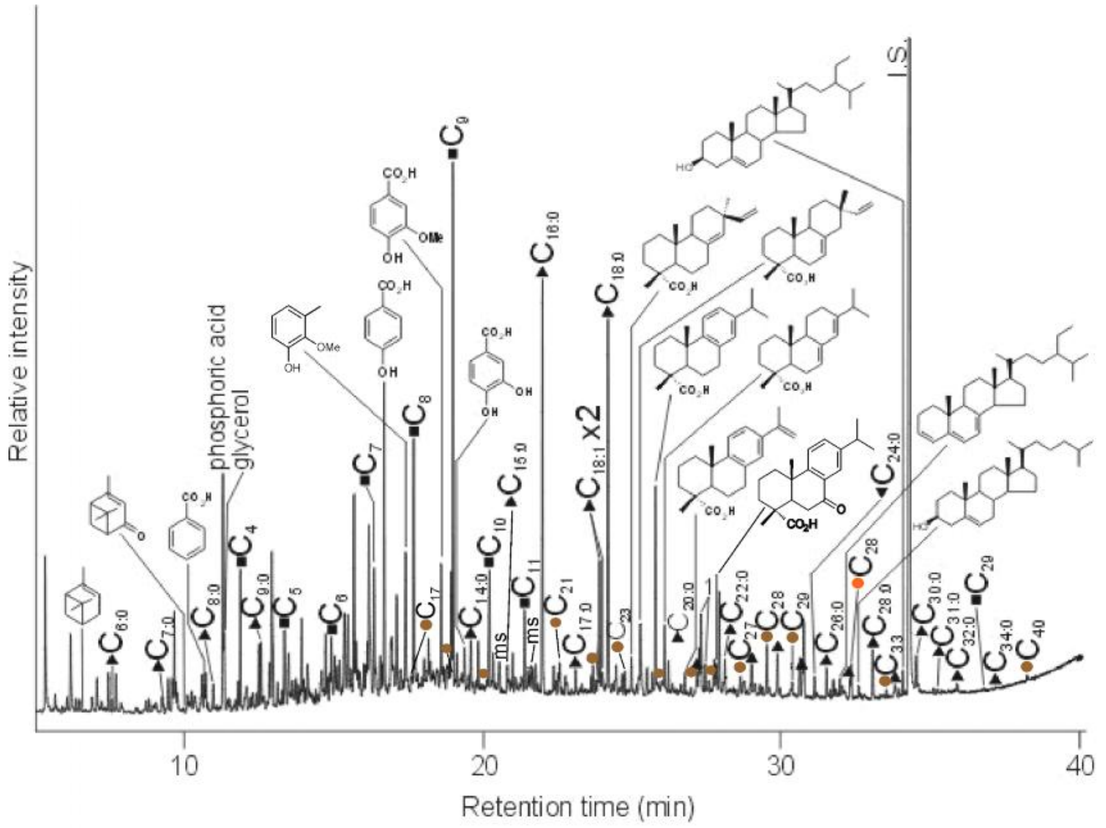

Figure S28 Reconstructed GC-MS TIC of the trimethylsilylated total lipid extract of 33.30.92 2 (see Table 2). Peak identities as for Fig. 3 and S14-S27. Also shown is the structure of the monoterpenoid, α-pinene (tentatively identified).

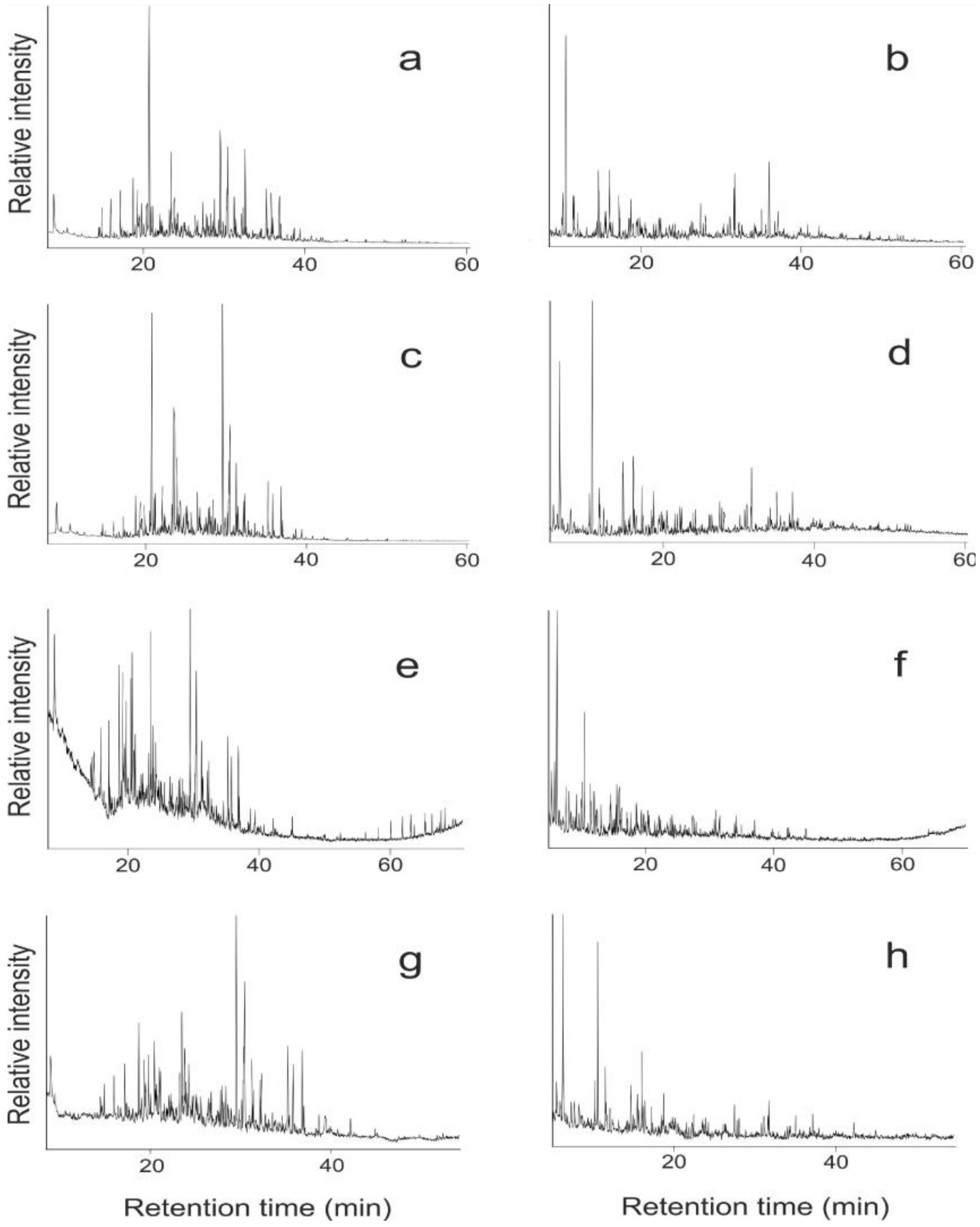

Figure S29 Reconstructed sequential TD-GC-MS and Py-GC-MS TICs of the 'resin' samples 33.30.44 2, 33.30.80 2, 33.30.83 2, 33.30.92 1: a, TD-GC-MS of sample 33.30.44 2; b, Py-GC-MS of sample 33.30.44 2; c, TD-GC-MS of sample 33.30.80 2; d, Py-GC-MS of sample 33.30.80 2; e, TD-GC-MS of sample 33.30.83 2; f, Py-GC-MS of sample 33.30.83 2; g, TD-GC-MS of sample 33.30.92 1; h, Py-GC-MS of sample 33.30.92 1.

Supplementary references:

1. Petrie WMF (1921) *Corpus of Prehistoric Pottery and Palettes*. (British School of Archaeology in Egypt and Bernard Quaritch, London).
2. Rost FWD, Oldfield RJ (2000) *Photography with a Microscope* (Cambridge Univ Press, Cambridge).
3. Oldfield RJ (1994) *Light Microscopy: an Illustrated Guide*. (Wolfe Publishing, London).
4. Dee M, Bronk Ramsey C (2000) Refinement of Graphite Target Production at ORAU. *Nucl Instrum Meth Phys Res B* 172: 449-453.
5. Bronk Ramsey C (2009) Bayesian analysis of radiocarbon dates. *Radiocarbon* 51: 337-360.
6. Stuiver M, Polach HA (1977) Discussion: Reporting of ^{14}C Data. *Radiocarbon* 19: 355-363.
7. Reimer, P.J., Bard, E., Bayliss, A., Beck, J.W., Blackwell, P.G., Bronk Ramsey, C., Grootes, P.M., Guilderson, T.P., Haflidason, H., Hajdas, I., Hatté, C., Heaton, T.J., Hoffmann, D.L., Hogg, A.G., Hughen, K.A., Kaiser, K.F., Kromer, B., Manning, S.W., Mu Niu, Reimer, R.W., Richards, D.A., Scott, E.M., Southon, J.R., Staff, R.A., Turney, C.S.M. and van der Plicht., J. 2013. IntCal13 and Marine13 Radiocarbon Age Calibration Curves 0–50,000 Years cal BP. *Radiocarbon* 55(4): 1869-1887.
8. Dee M, Wengrow D, Shortland A, Stevenson A, Brock F, Girdland Flink L, Ramsey Christopher (2013) An absolute chronology for early Egypt using radiocarbon dating and Bayesian statistical modelling. Proc R Soc A 469: 20130395 - 4th September 2013 (doi: 10.1098/rspa.2013.0395)
9. Hassan FA (1985) Radiocarbon Chronology of Neolithic and Predynastic sites in Upper Egypt and the Delta. *African Archaeological Review* 3: 95-116.
10. Hendrickx S (2006) in *Ancient Egyptian Chronology. Handbook of Oriental Studies*, eds Hornung E, Krauss R, Warburton DA (Brill, Leiden/Boston), pp 55-93
11. Buckley SA, Stott AW, Evershed RP (1999) Studies of organic residues from ancient Egyptian mummies using high temperature-gas chromatography-mass spectrometry and sequential thermal desorption-gas chromatography-mass spectrometry and pyrolysis-gas chromatography-mass spectrometry. *Analyst* 124: 443-452.
12. van den Berg KJ, Boon JJ, Pastorova I, Spetter LFM (2000) Mass spectrometric methodology for the analysis of highly oxidized diterpenoid acids in Old Master paintings. *J Mass Spectrom* 35(4): 512-533.
13. Stacey RJ, Cartwright CR, McEwan C (2006) Chemical characterization of ancient Mesoamerican 'copal' resins: preliminary results. *Archaeometry* 48(2): 323-340.
14. Colombini MP, Modugno F, Ribechini E (2005) Direct exposure electron ionization mass spectrometry and gas chromatography/mass spectrometry techniques to study organic coatings on archaeological amphorae. *J Mass Spectrom* 40: 675-687.
15. Steigenberger G, Herm C (2011) Natural resins and balsams from an eighteenth-century pharmaceutical collection analysed by gas chromatography/mass spectrometry. *Anal and Bioanal Chem* 401: 1771-1784.
16. Hansen SL, Krueger WJ, Dunn Jr. LB, Artz WE (1997) Nuclear Magnetic Resonance and Gas Chromatography/Mass Spectroscopy Analysis of the Nonvolatile Components Produced during Heating of Oleic Acid Esterified Propoxylated Glycerol, a Fat Substitute Model Compound, and Trioleylglycerol. *J Agric Food Chem* 45(12): 4730-4739.
17. Christopoulou CN, Perkins EG (1989) Dimer Acids: Synthesis and Mass Spectrometry of the Tetrahydroxy, Dihydroxy, and Diketo Dimers of Methyl Stearate. *J American Oil Chemists Society* 66(9): 1344-1352.
18. Brooks CJW, Horning EC, Young JS (1968) Characterization of Sterols by Gas Chromatography-Mass Spectrometry of the Trimethylsilyl Ethers. *Lipids* 3(5): 391-402.
19. Rontani J-F, Aubert C (2005) Electron ionization mass spectral fragmentation of cholestane-3β,4α,5α-triol and cholestane-3β,5α,6α/β-triol bis- and tris-trimethylsilyl derivatives. *Rapid Commun Mass Spectrom* 19: 1921-1927.
20. Grandgirard A, Martine L, Joffre C, Juaneda P, Berdeaux O (2004) Gas chromatographic separation and mass spectrometric identification of mixtures of oxyphytosterol and

oxycholesterol derivatives: Application to a phytosterol-enriched food. *J Chromatogr A* 1040: 239-250.
21. Pulfer MK, Taube C, Gelfand E, Murphy RC (2005) Ozone Exposure in Vivo and Formulation of Biologically Active Oxysterols in the Lung. *J Pharmacol Exp Ther* 312(1): 256-264.
22. Beste L, Nahar N, Dalman K, Fujioka S, Jonsson L (2011) Synthesis of Hydroxylated Sterols in Transgenic Arabidopsis Plants Alters Growth and Steroid Metabolism. *Plant Physiol* 157: 426-440.
23. Gray MF, Lawrie TDV, Brooks CJW (1972) Isolation and Identification of Cholesterol α–Oxide and Other Minor Sterols in Human Serum. *Lipids* 6(11): 836-843.
24. Björkhem I, Breuer O, Angelin B, Wikström S-Å (1988) Assay of unesterified cholesterol-5,6-epoxide in human serum by isotope dilution mass spectrometry. Levels in the healthy state and in hyperlipoproteinemia. *J Lipid Res* 29: 1031-1038.
25. Madaio A, Notaro G, Piccialli V, Sica D (1990) Minor 5,6-secosterols from the marine sponge *Hippospongia communis*. Isolation and synthesis of (7*Z*,22*E*,24*R*)-24-methyl-5,6-secocholesta-7,22-diene-3β,5β,6-triol. *J Nat Prod* 53(3): 565-572.
26. Madaio A, Piccialli V, Sica D (1989) New polyhydroxysterols from the dictyoceratid sponges *Hippospongia communis*, *Spongia officinalis*, *Ircinia variabilis*, and *Spongionella gracilis*. *J Nat Prod* 52(5): 952-961.
27. Migliuolo A, Piccialli V, Sica D, Giordano F (1993) New Δ^{8}- and $\Delta^{8(14)}$-5α,6α-epoxysterols from the marine sponge *Spongia officinalis*. *Steroids* 58: 134-140.
